# Supplementary material for: Growth Hormone Upregulates Melanoma Drug Resistance and Migration via Melanoma-Derived Exosomes
Source: Cancers (Basel). 2024 Jul 24;16(15):2636. doi: 10.3390/cancers16152636 (PMC11311539; doi:10.3390/cancers16152636)
Supplement: Supplementary file 1 [file cancers-16-02636-s001.zip › cancers-3099853-supplementary/cancers-3099853-supplementary.pdf]

Supplementary data

Growth Hormone upregulates melanoma drug resistance and migration via melanoma derived exosomes

Prateek Kulkarni[1,2,3], Reetobrata Basu[1], Taylor Bonn[1,4], Beckham Low[1,3], Nathaniel Mazurek[1,5], and John J Kopchick[1,2,6 *]

1. Edison Biotechnology Institute, Ohio University, Athens, OH 45701
2. Molecular and Cellular Biology Program, Ohio University, Athens, OH 45701
3. Department of Biological Sciences, Ohio University, Athens, OH 45701
4. Department of Nutrition Ohio University, Athens, OH 45701
5. Environmental and Plant Biology Ohio University, Athens, OH 45701
6. Department of Biomedical Sciences Ohio University, Athens, OH 45701

*Correspondence: kopchick@ohio.edu; Tel.: +1 740-593-4534

Supplementary Figure 1.

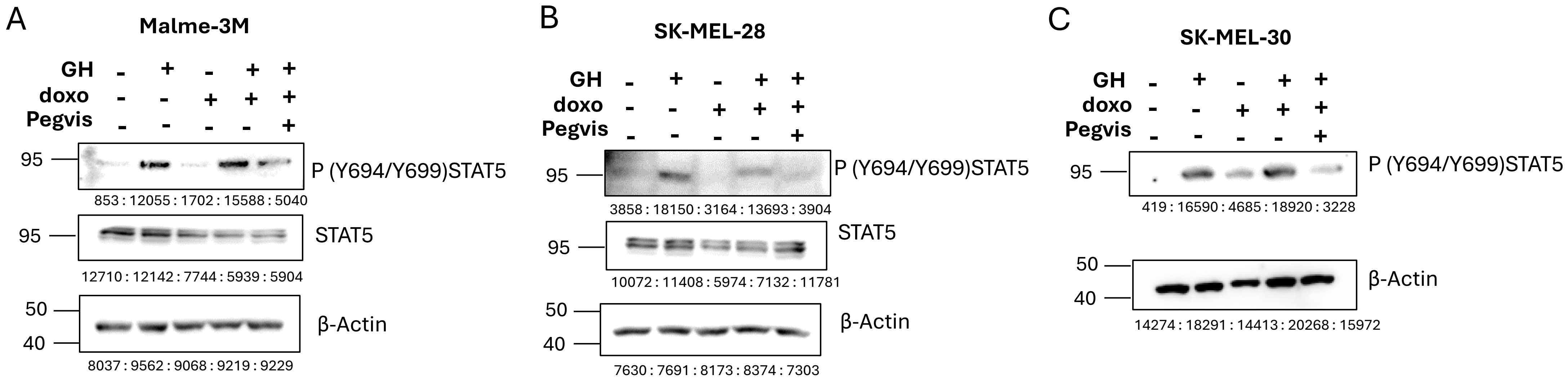

Figure S1: GH promotes and GHR antagonist, pegvisomant, attenuates phosphorylation levels of STAT5. Protein levels of (Y694/Y699) STAT5, STAT5, and β-actin in human melanoma cells, Malme-3M, SK-Mel-28, and SK-MEL-30, 10 minutes post treatment with 50 ng/ml GH independently or in combination with doxorubicin and 500 nM pegvisomant.

Supplementary Figure 2.

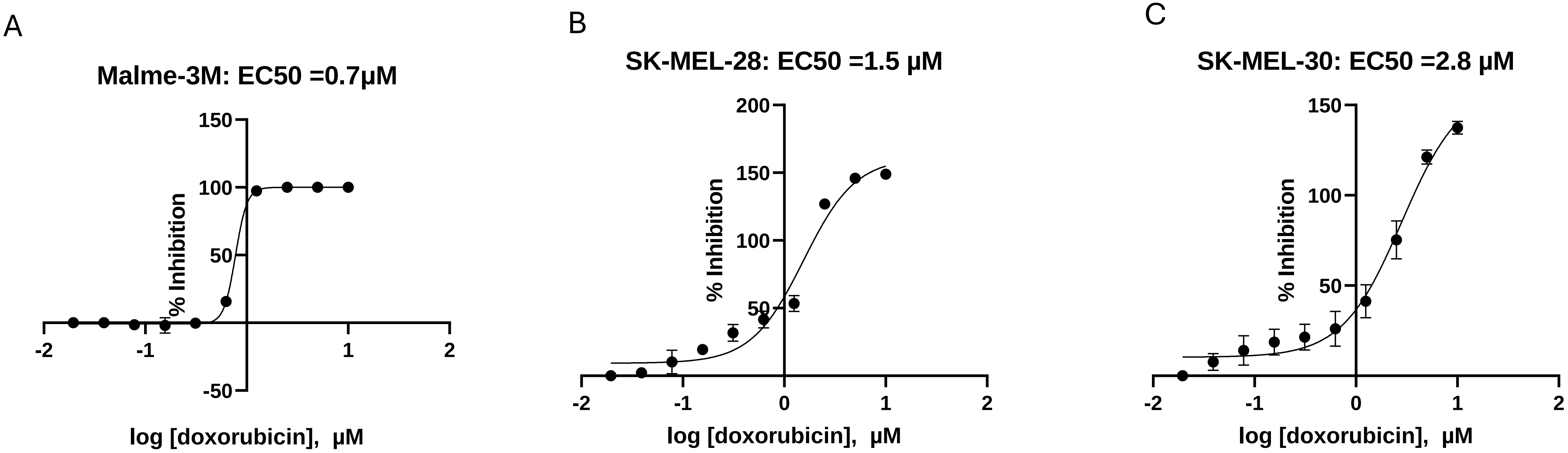

Figure. S2: EC50s (in μM) of the doxorubicin chemotherapy in (A) Malme-3M, (B) SK-MEL-28, (C) SK-MEL-30 human melanoma cell lines. Resazurin-resorufin cell viability assay (48-hr) was employed for 10-dose EC50 determination in 96-well plate format with 5000 cells/well. Percent inhibition was calculated and nonlinear regression (variable slope – four parameters) was used to calculate EC50 in Graphpad Prizm.

Supplementary Figure 3.

A

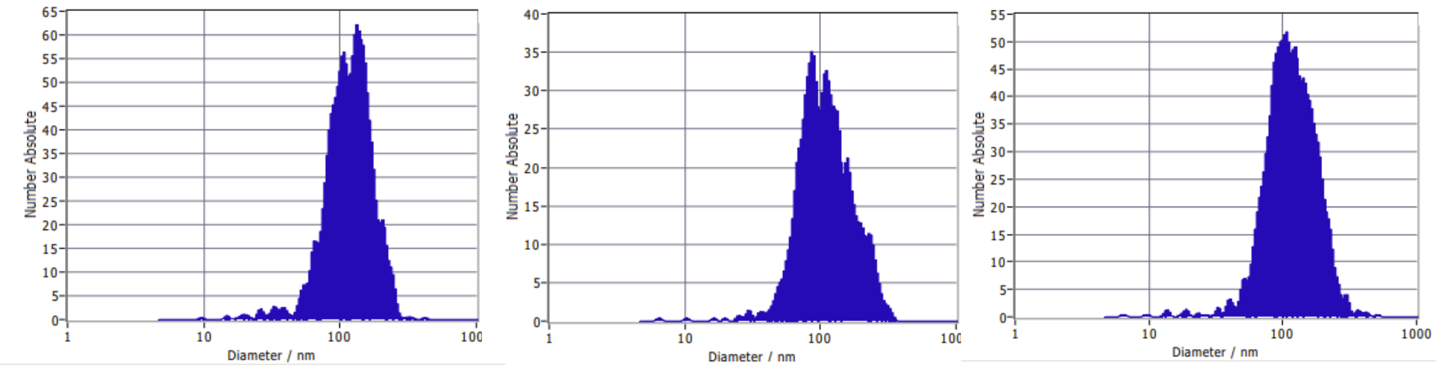

	Malme-3M	SK-MEL-28	SK-MEL-30
Peak diameter (nm)	106.5	110.7	104.1
Concentration (Particles / ml)	1.8×10^{11}	1.4×10^{11}	1.4×10^{11}

B

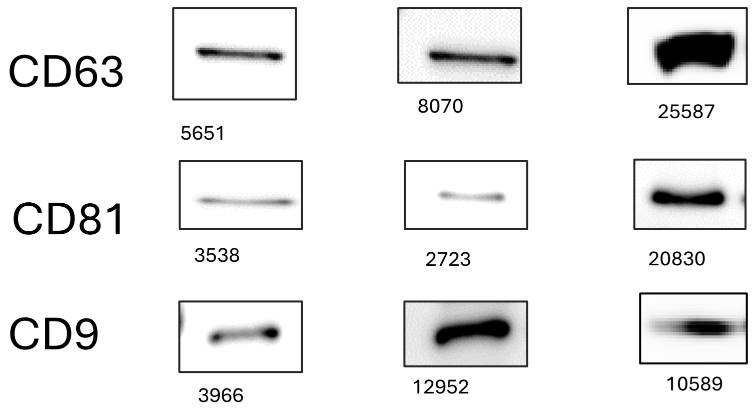

C

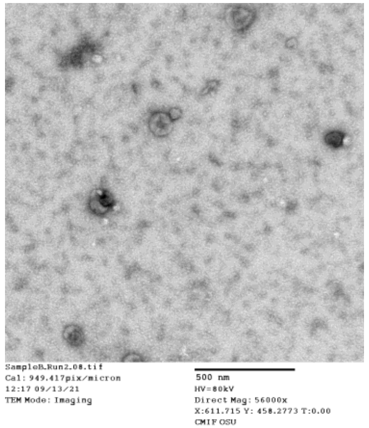

FigureS3: **Characterization of melanoma-derived exosomes**. A. Nanoparticle Tracking Analysis of exosomes. B. Exosome markers CD63, CD9, CD81 as detected by western blot. C. Transmission electron microscope

Supplementary Figure 4.

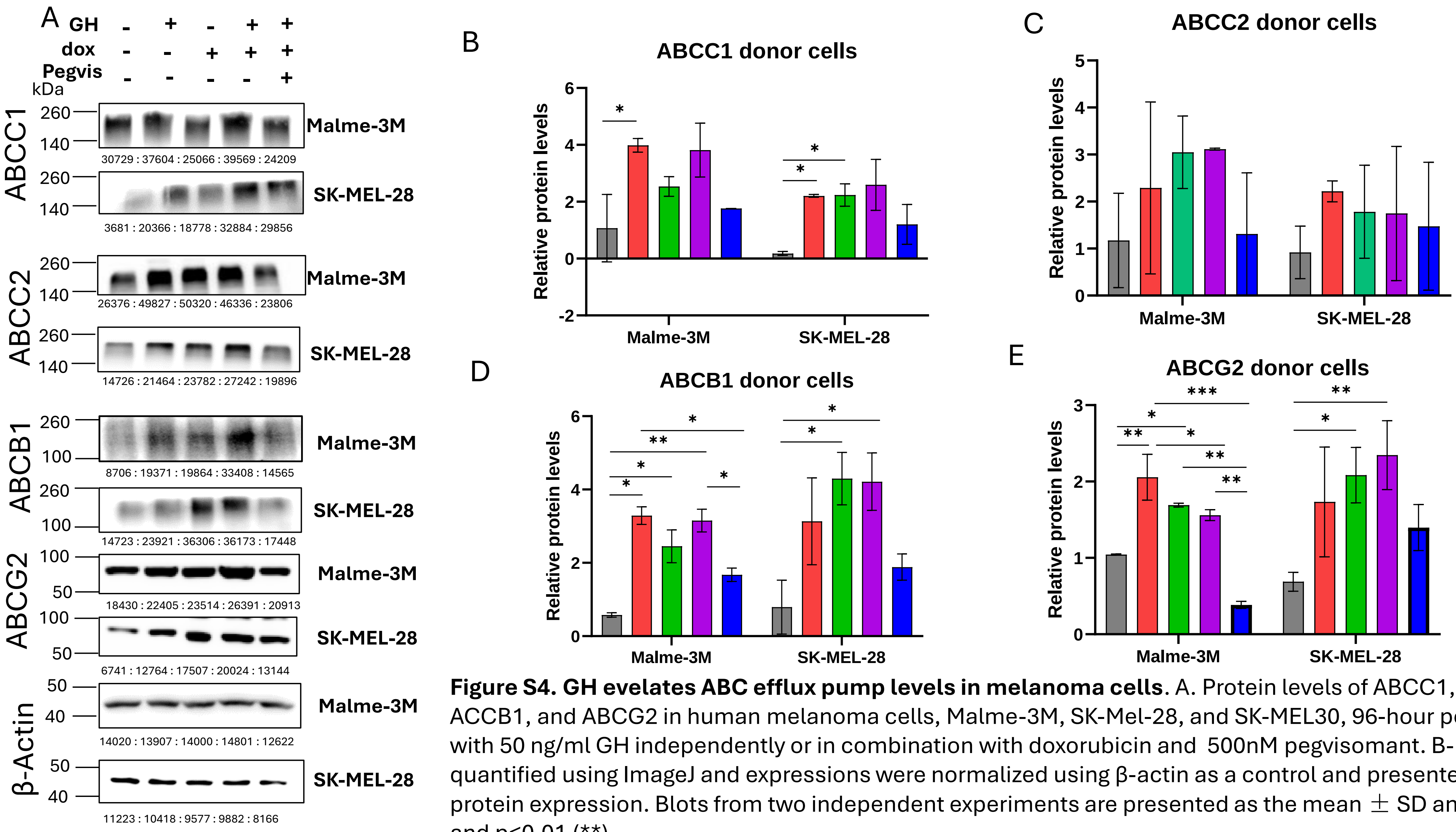

Figure S4. GH evelates ABC efflux pump levels in melanoma cells. A. Protein levels of ABCC1, ABCC2, ACCB1, and ABCG2 in human melanoma cells, Malme-3M, SK-Mel-28, and SK-MEL30, 96-hour post treatment with 50 ng/ml GH independently or in combination with doxorubicin and 500nM pegvisomant. B-E. Blots were quantified using ImageJ and expressions were normalized using β-actin as a control and presented as relative protein expression. Blots from two independent experiments are presented as the mean ± SD and p <0.05 (*), and p<0.01 (**).

Supplementary Figure 5.

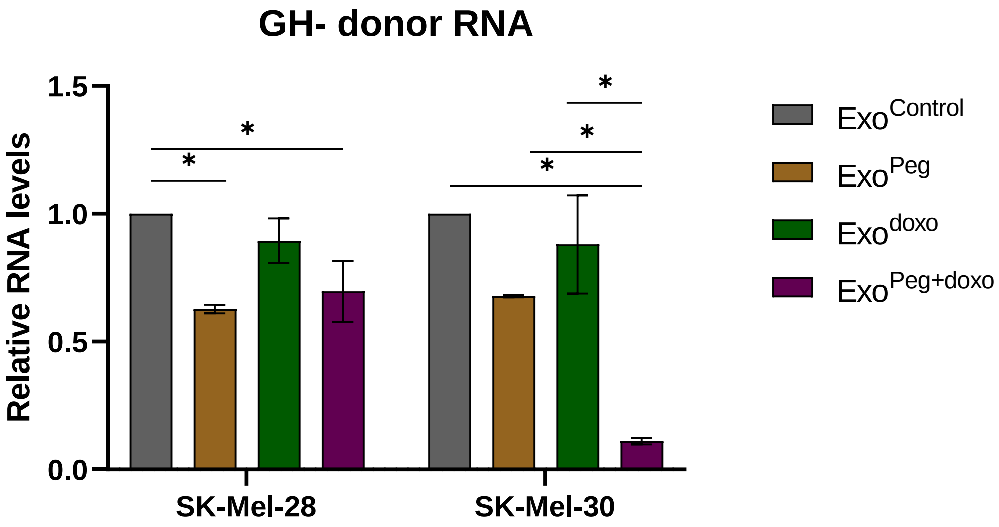

Figure S5. Pegvisomant downregulates basal levels of GH in human melanoma cells in SK-Mel-28, and SK-MEL30, 24-hour post treatment with EC50 dosage of doxorubicin or in combination with 500nM pegvisomant.

Supplementary Figure 6.

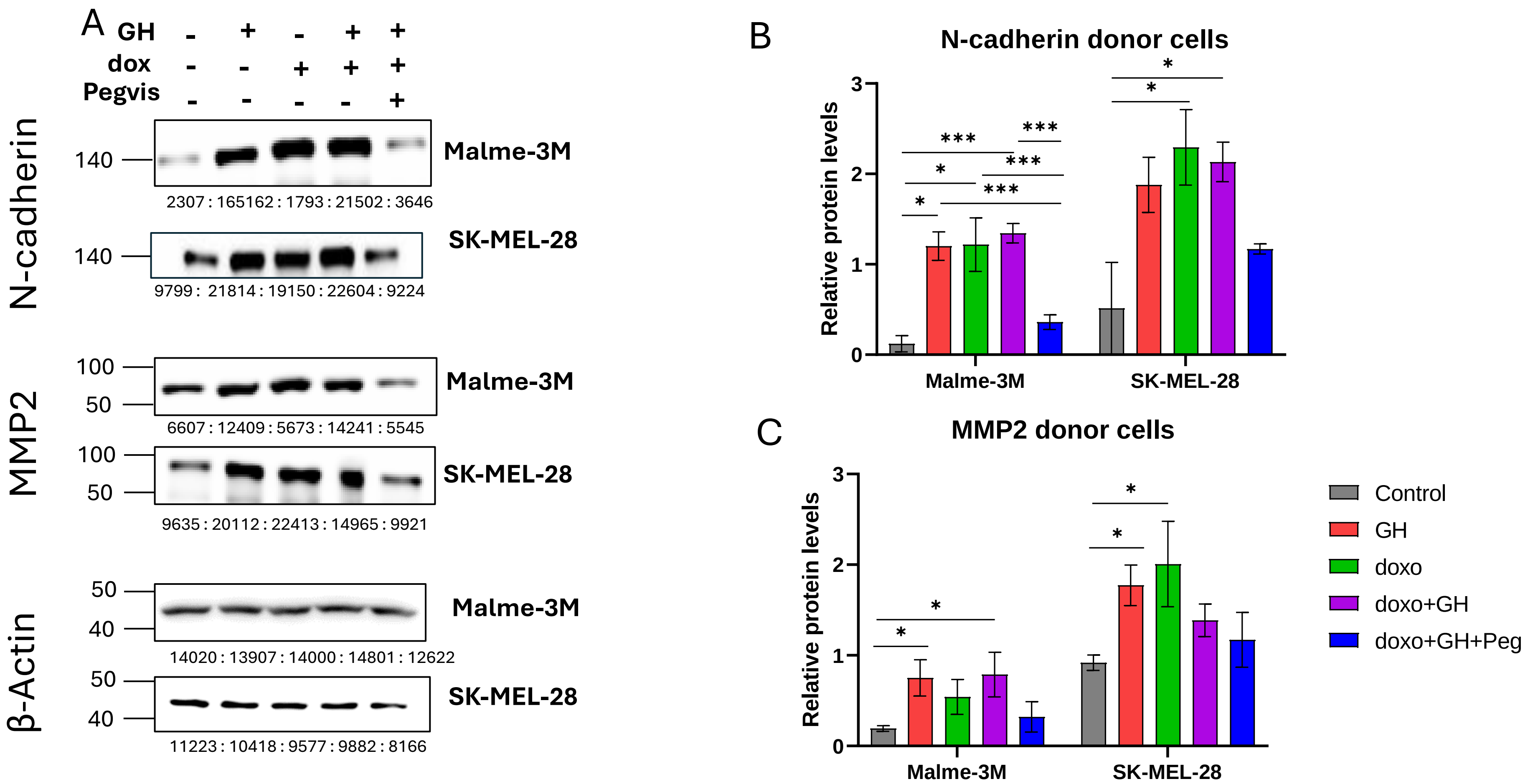

Figure S6. GH elevates ABC efflux pump levels in melanoma cells. A. Protein levels of ABCC1, ABCC2, ACCB1, amd ABCG2 in human melanoma cells, Malme-3M, SK-Mel-28, and SK-MEL30, 96-hour post treatment with 50 ng/ml GH independently or in combination with EC50 dosage of doxorubicin, 250nM pegvi-somant. B-E. Blots were quantified using ImageJ and expressions were normalized using β-actin as a contorl and presented as relative protein expression. Blots from two independent experiments are presented as the mean ± SD and p <0.05 (*), p<0.01 (**), and p<0.001 (***).

Supplementary Figure 7.

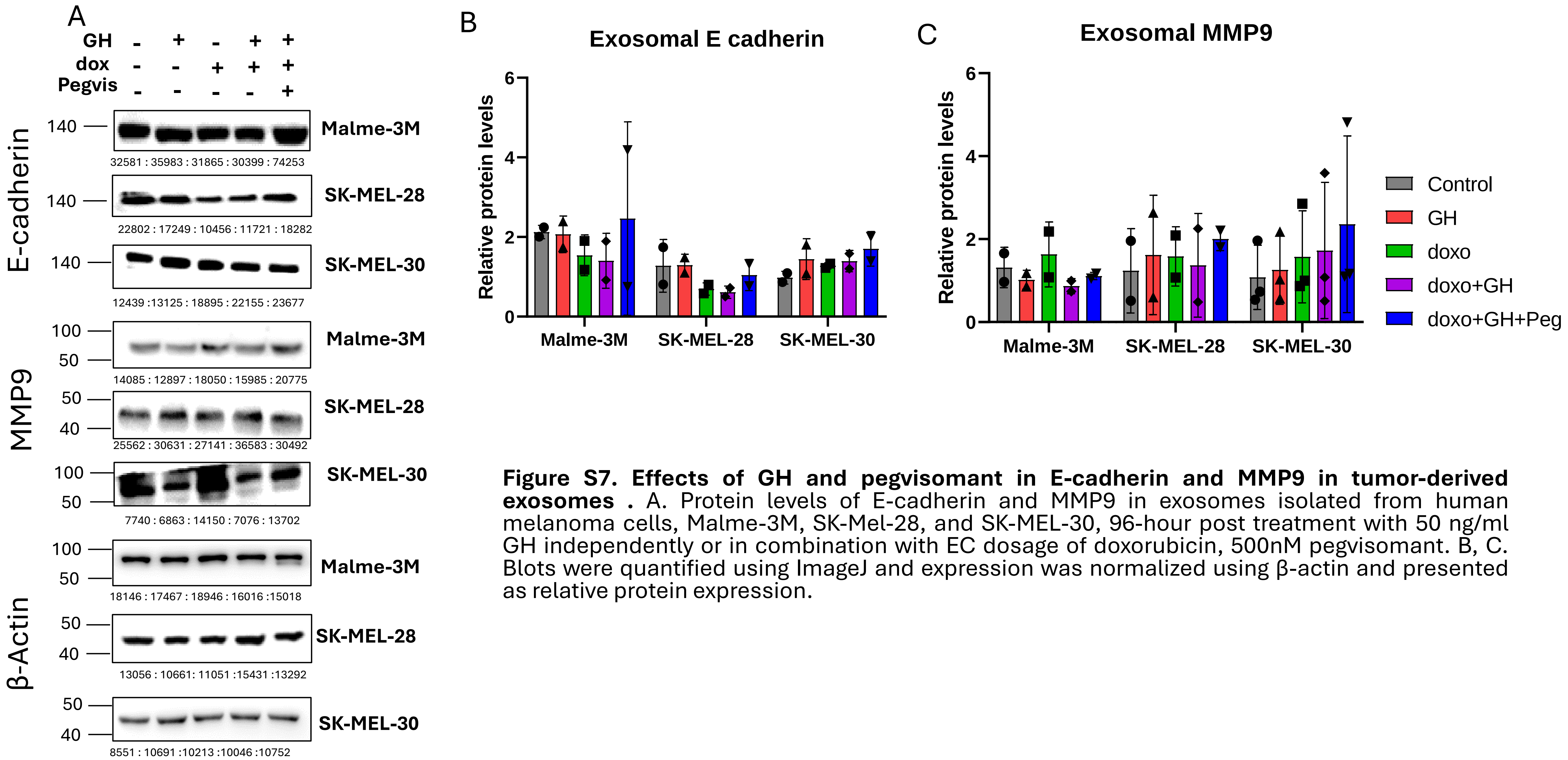

Figure S7. Effects of GH and pegvisomant in E-cadherin and MMP9 in tumor-derived exosomes . A. Protein levels of E-cadherin and MMP9 in exosomes isolated from human melanoma cells, Malme-3M, SK-Mel-28, and SK-MEL-30, 96-hour post treatment with 50 ng/ml GH independently or in combination with EC dosage of doxorubicin, 500nM pegvisomant. B, C. Blots were quantified using ImageJ and expression was normalized using β-actin and presented as relative protein expression.

Supplementary Figure 8.

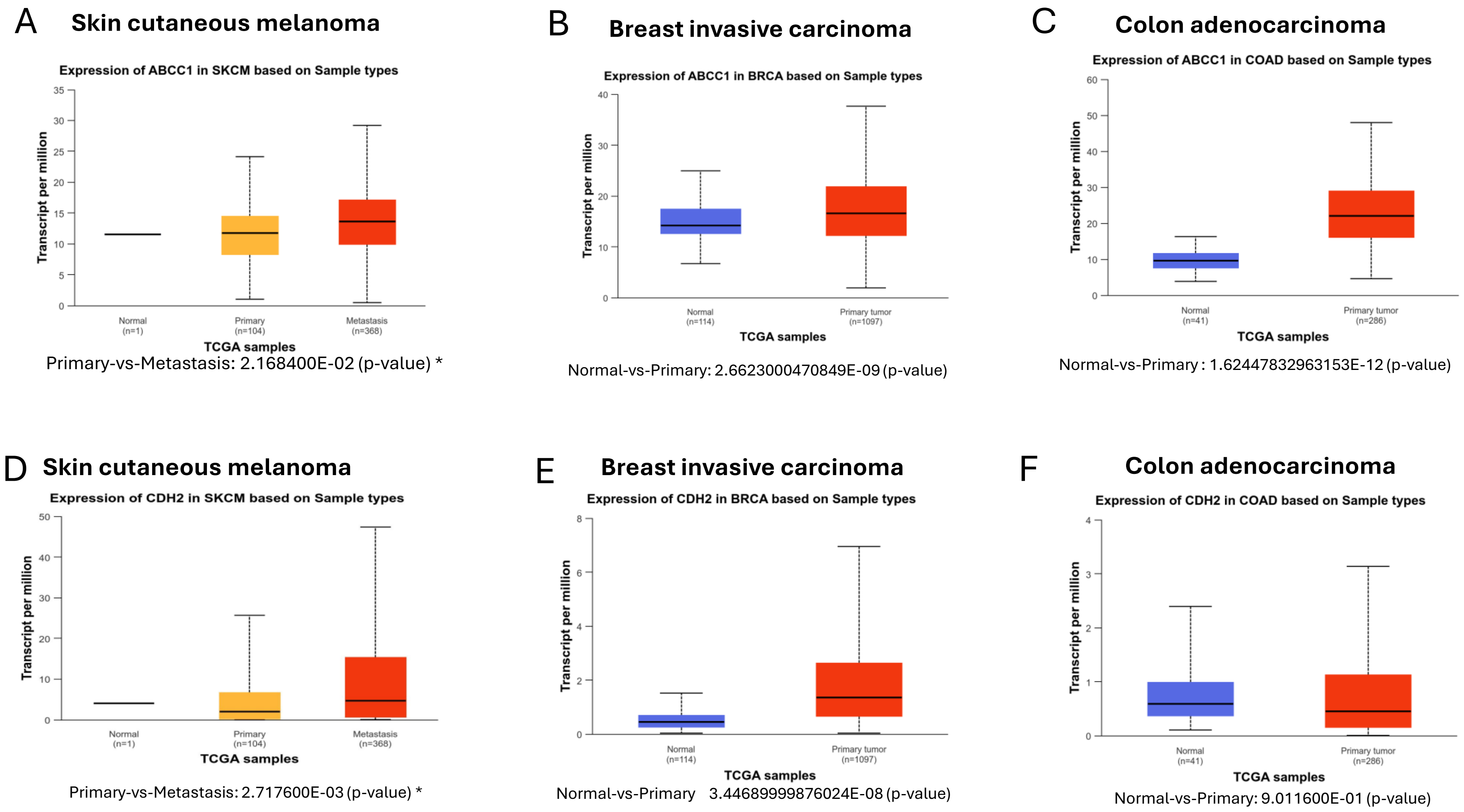

Figure S8. Expression levels of ABCC1 and N-acdherin (CDH2) mRNA in TCGA dataset assessed in UALCAN webserver in (A, D) Skin cutaneous melanoma (SKCM), (B,E) breast invasive carcinoma (BRCA), and (C, F) Colon adenocarcinoma (COAD)
