# Supplementary material for: Growth Hormone Upregulates Melanoma Drug Resistance and Migration via Melanoma-Derived Exosomes
Source: Cancers (Basel). 2024 Jul 24;16(15):2636. doi: 10.3390/cancers16152636 (PMC11311539; doi:10.3390/cancers16152636)

Raw images (uncropped) images and no picture correction for western blot images

Growth Hormone upregulates melanoma drug resistance and migration via melanoma derived exosomes

Prateek Kulkarni[1,2,3], Reetobrata Basu[1], Taylor Bonn[1,4], Beckham Low[1,3], Nathaniel Mazurek[1,5], and John J Kopchick[1,2,6] *

1. Edison Biotechnology Institute, Ohio University, Athens, OH 45701
2. Molecular and Cellular Biology Program, Ohio University, Athens, OH 45701
3. Department of Biological Sciences, Ohio University, Athens, OH 45701
4. Department of Nutrition Ohio University, Athens, OH 45701
5. Environmental and Plant Biology Ohio University, Athens, OH 45701
6. Department of Biomedical Sciences Ohio University, Athens, OH 45701

*Correspondence: kopchick@ohio.edu; Tel.: +1 740-593-4534

Includes repeats and the sequence of lanes is from left to right

★ Star-marked images are used as representative image in the main figure

Western Blot Note: Verification of antibody specificity was previously performed across the entire blot (data not shown) to identify the specific molecular weights at which the bands appeared. For each experiment, the membrane was subsequently sectioned with adequate spacing at appropriate sizes to ensure safe handling during the western blot procedure. This was followed by primary antibody incubation and subsequent detection steps.

Figure 2. GH elevates the expression of ABC-transporter pumps in tumor-derived exosomes and their transfer to recipient cells

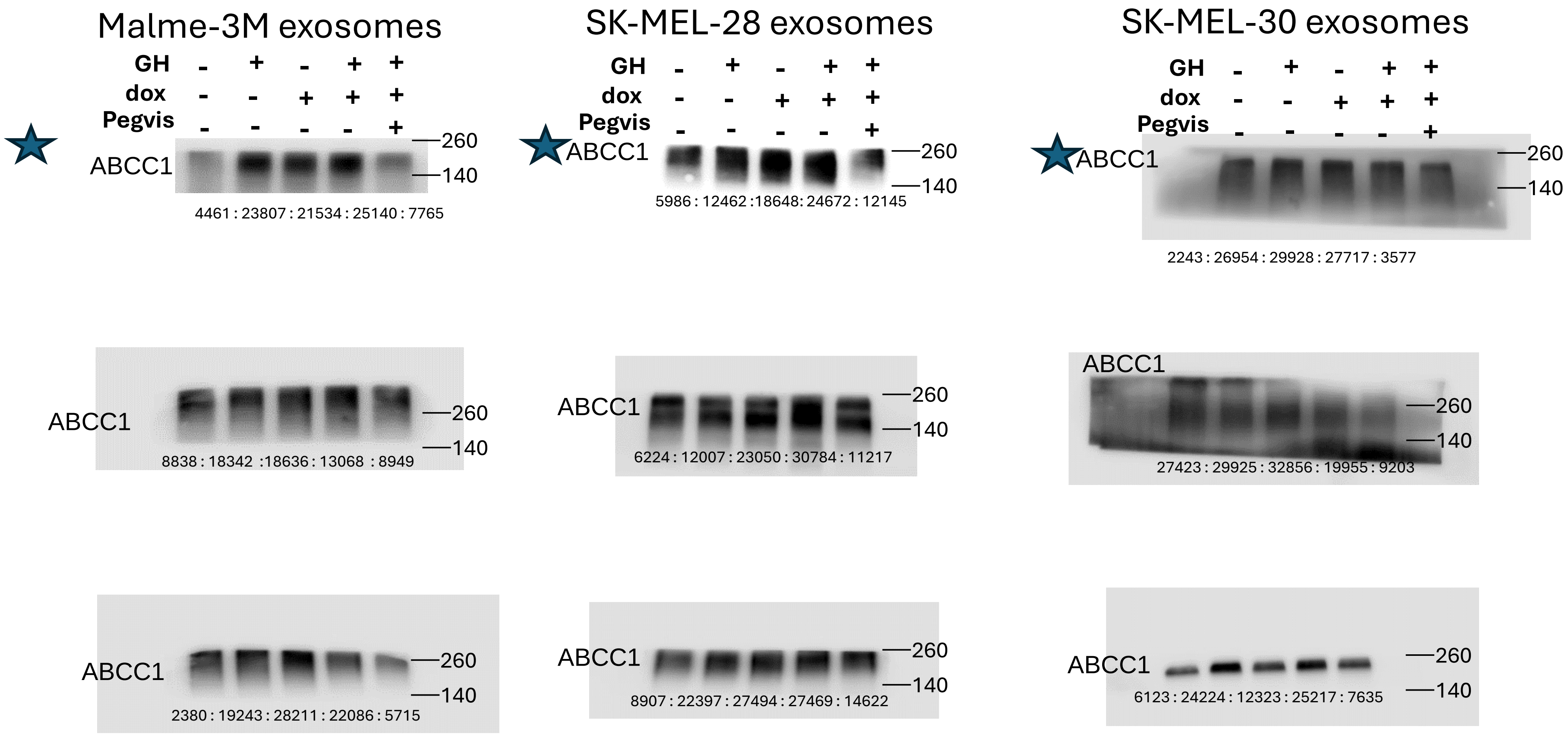

Figure 2. GH elevates the expression of ABC-transporter pumps in tumor-derived exosomes and their transfer to recipient cells

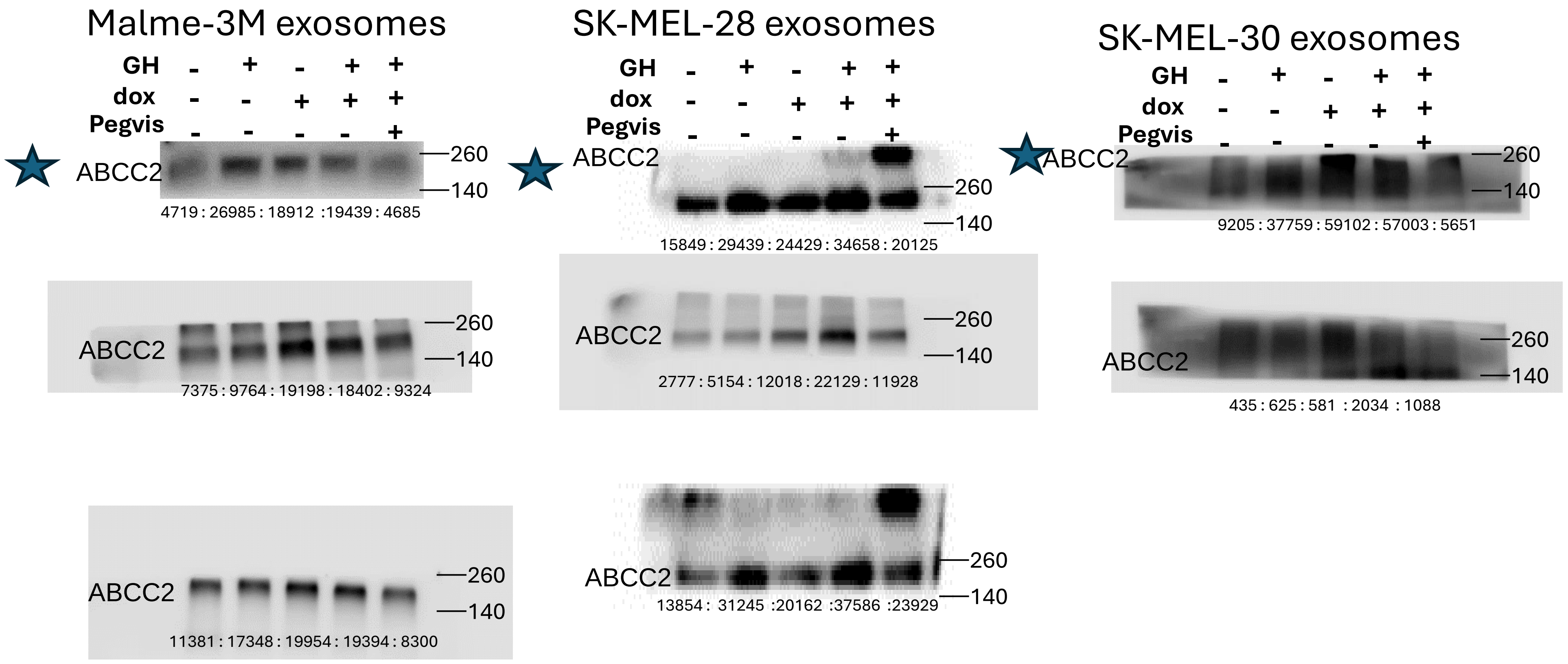

Figure 2. GH elevates the expression of ABC-transporter pumps in tumor-derived exosomes and their transfer to recipient cells

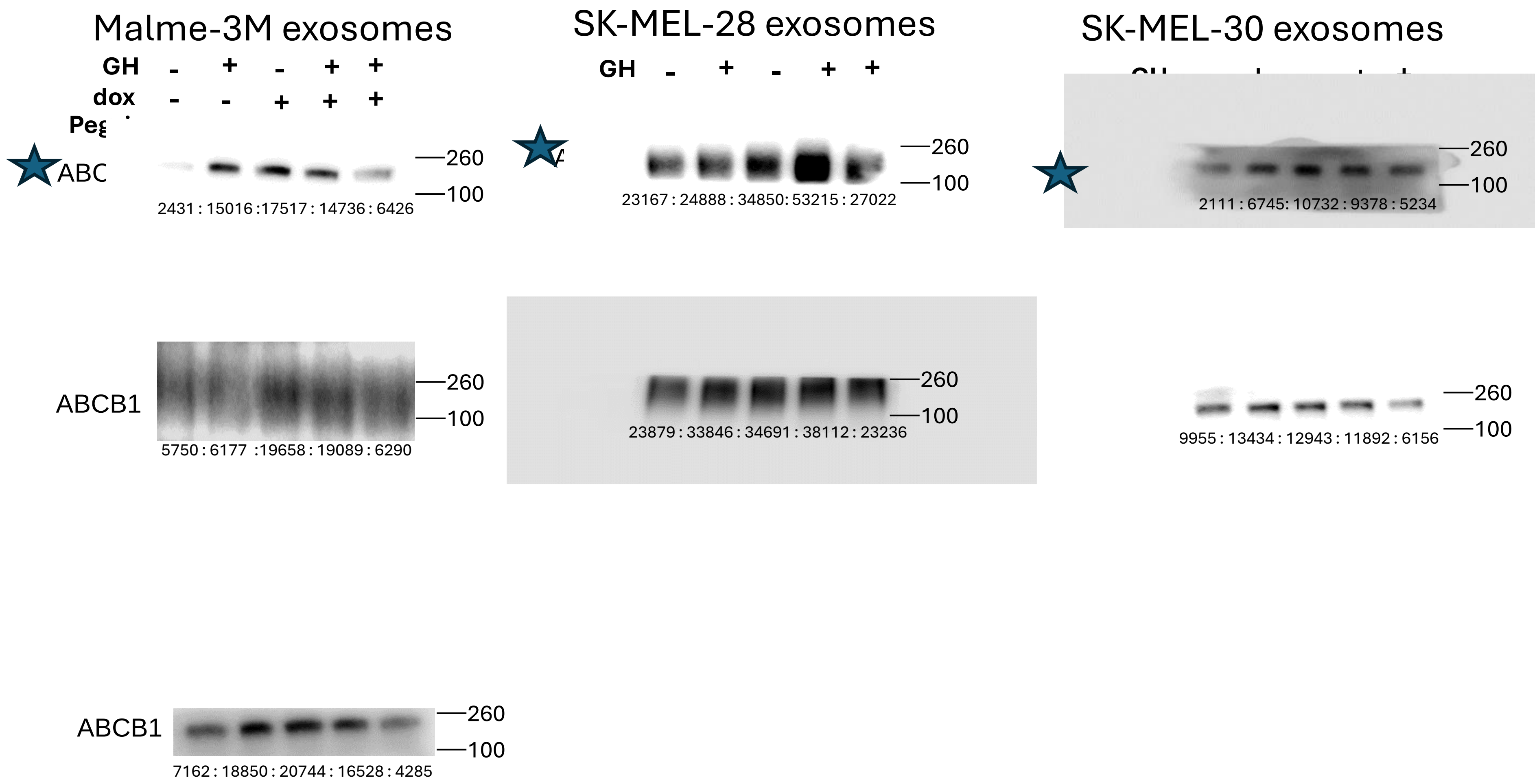

Figure 2. GH elevates the expression of ABC-transporter pumps in tumor-derived exosomes and their transfer to recipient cells

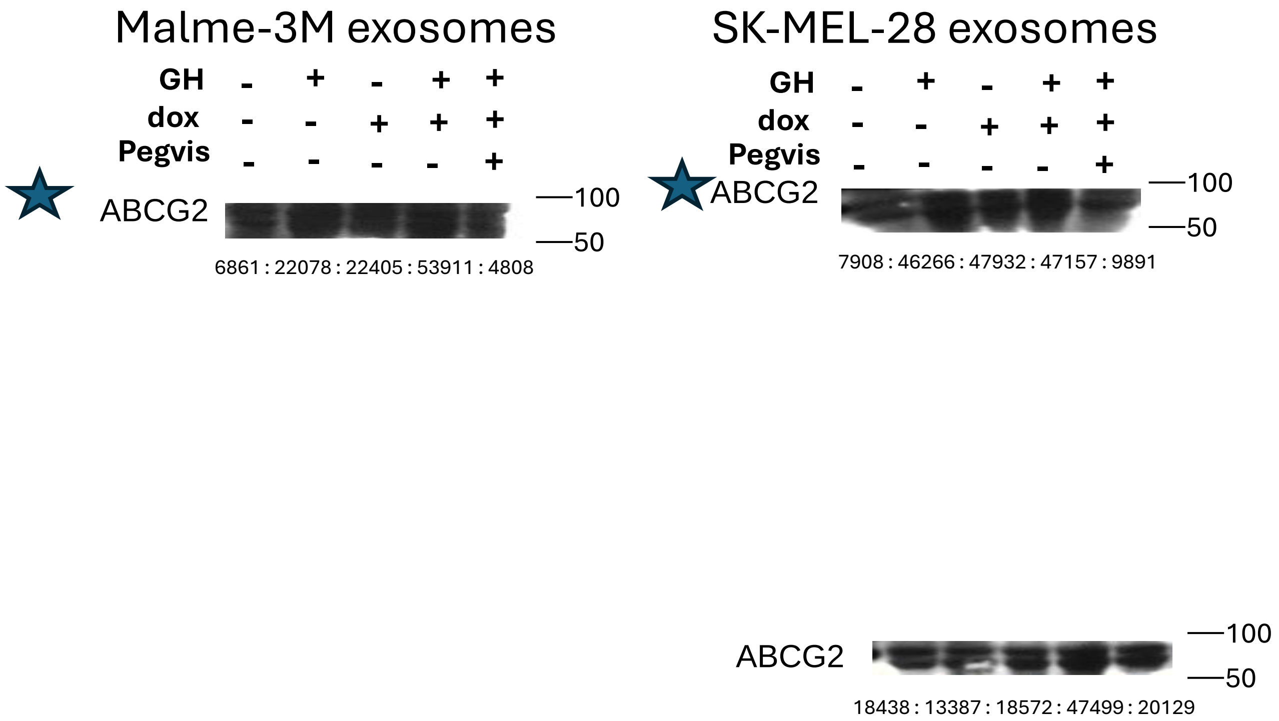

Figure 2. GH elevates the expression of ABC-transporter pumps in tumor-derived exosomes and their transfer to recipient cells

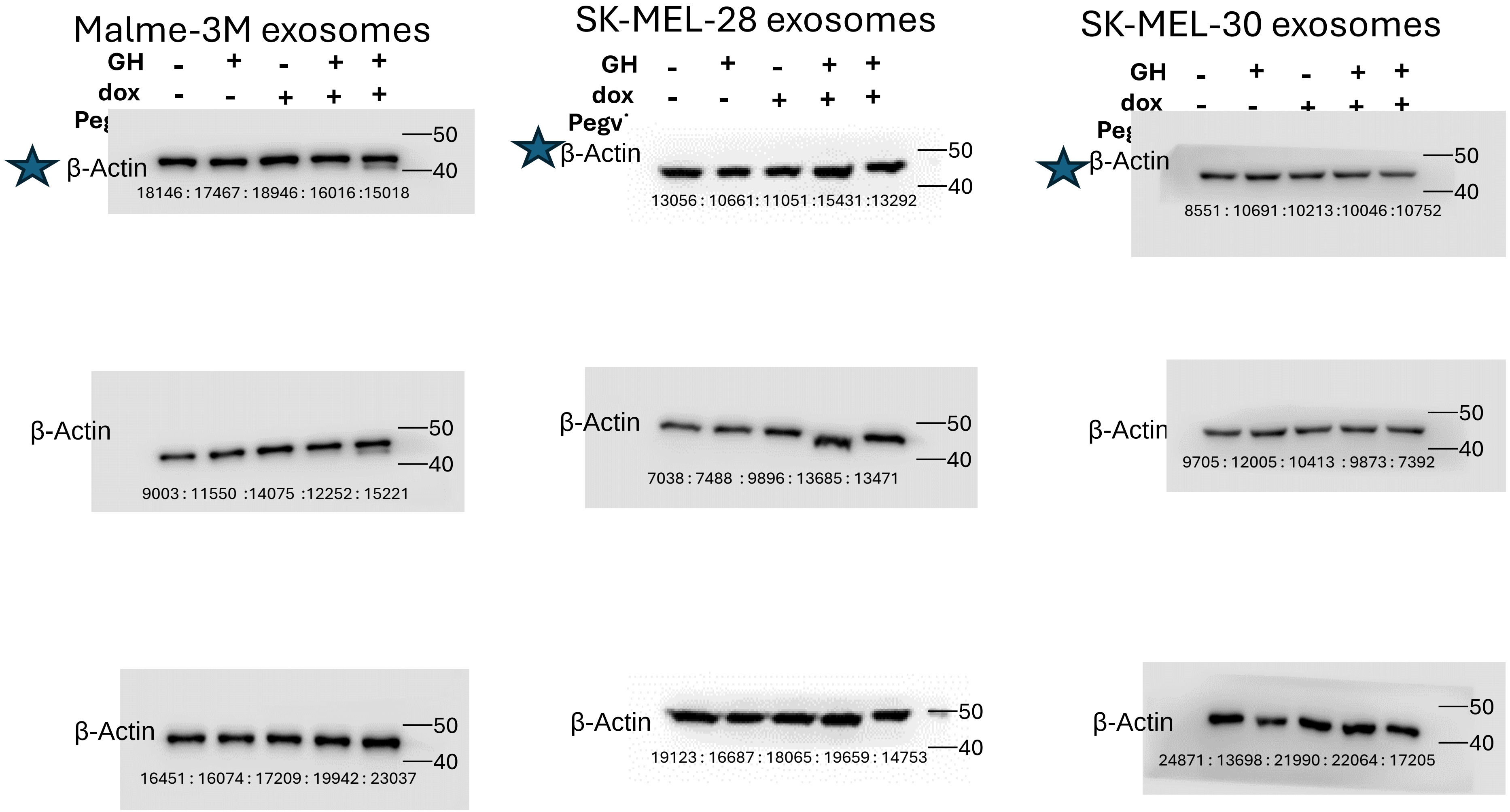

Figure 2. GH elevates the expression of ABC-transporter pumps in tumor-derived exosomes and their transfer to recipient cells

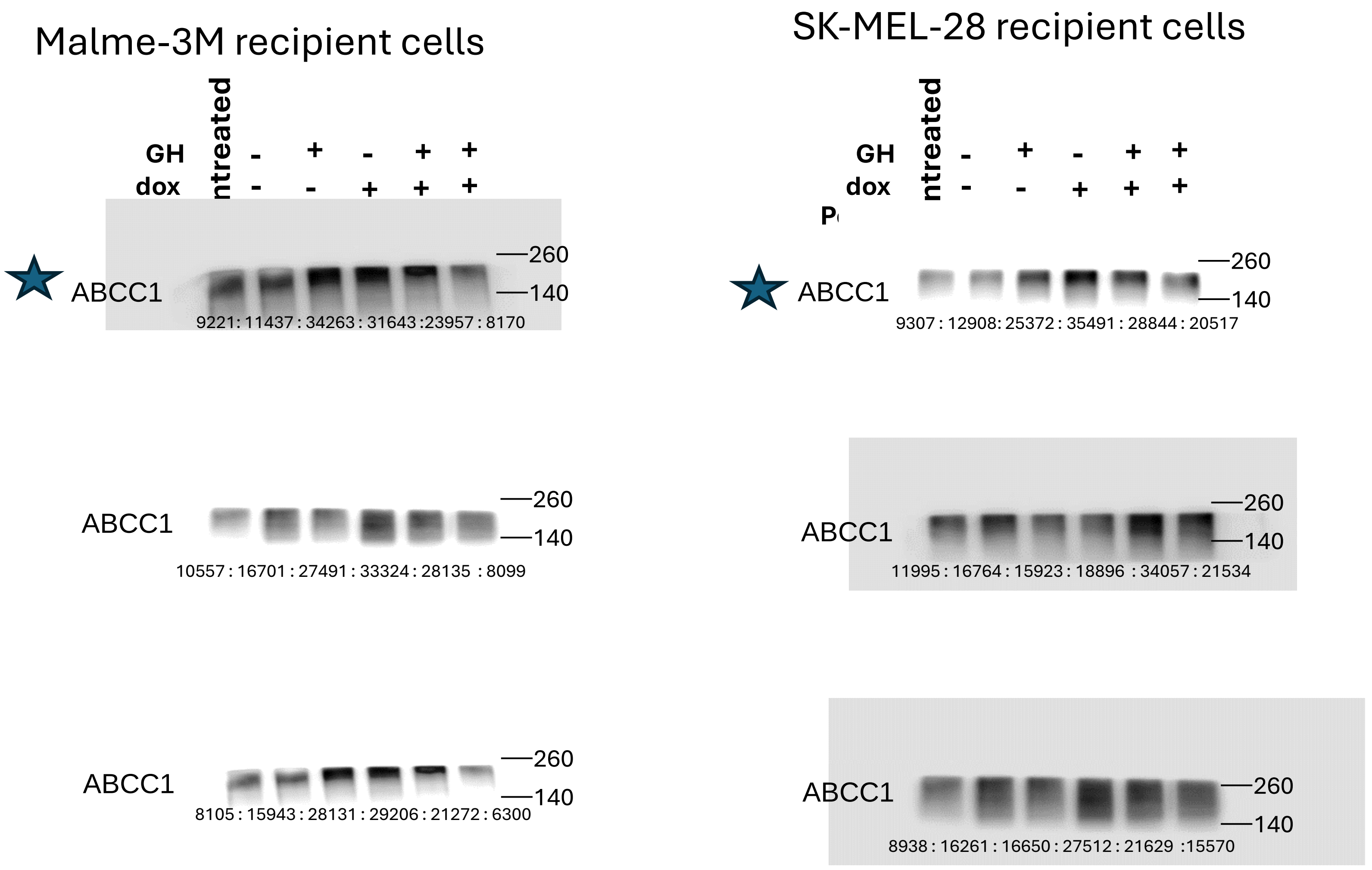

Figure 2. GH elevates the expression of ABC-transporter pumps in tumor-derived exosomes and their transfer to recipient cells

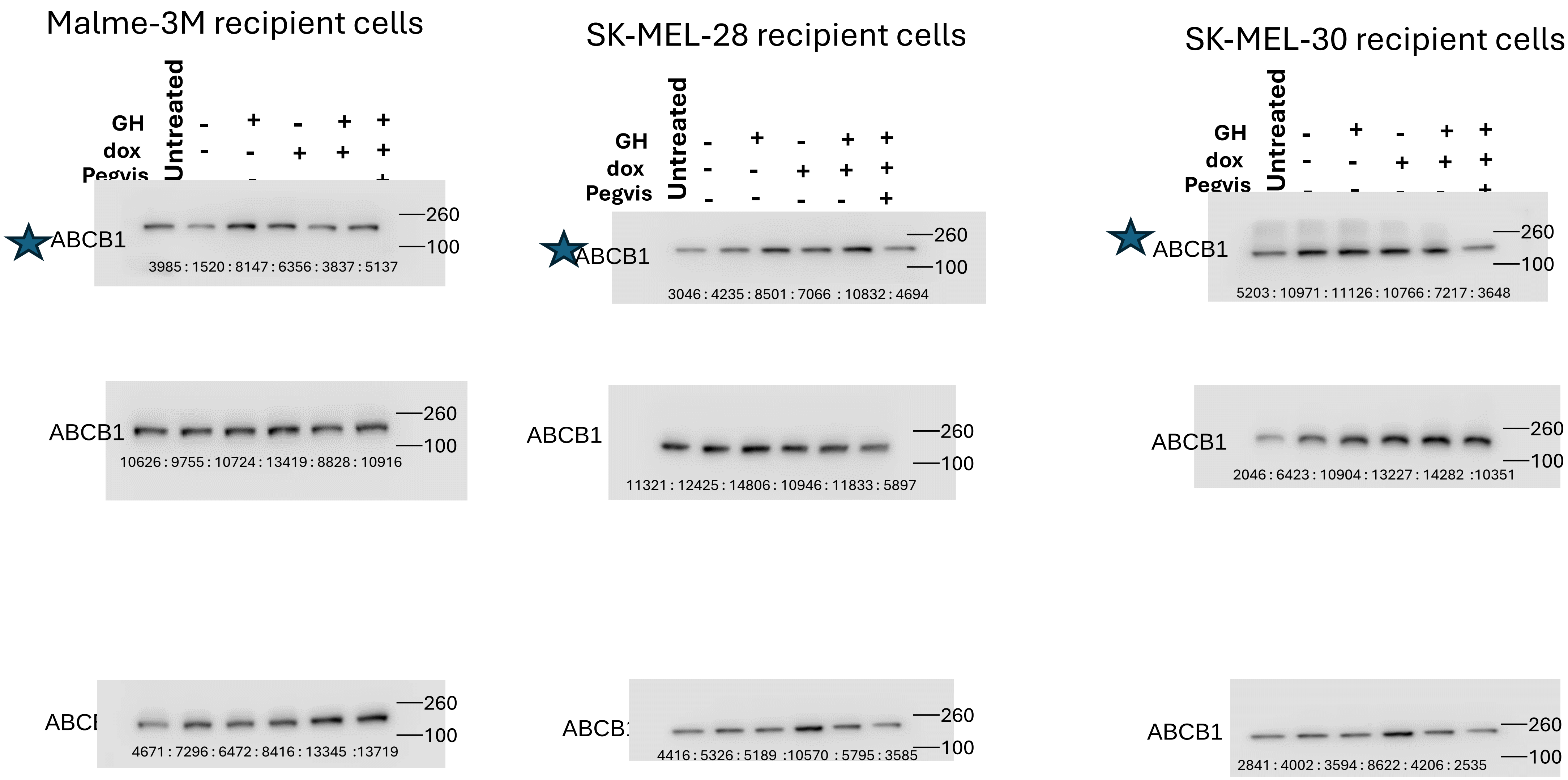

Figure 2. GH elevates the expression of ABC-transporter pumps in tumor-derived exosomes and their transfer to recipient cells

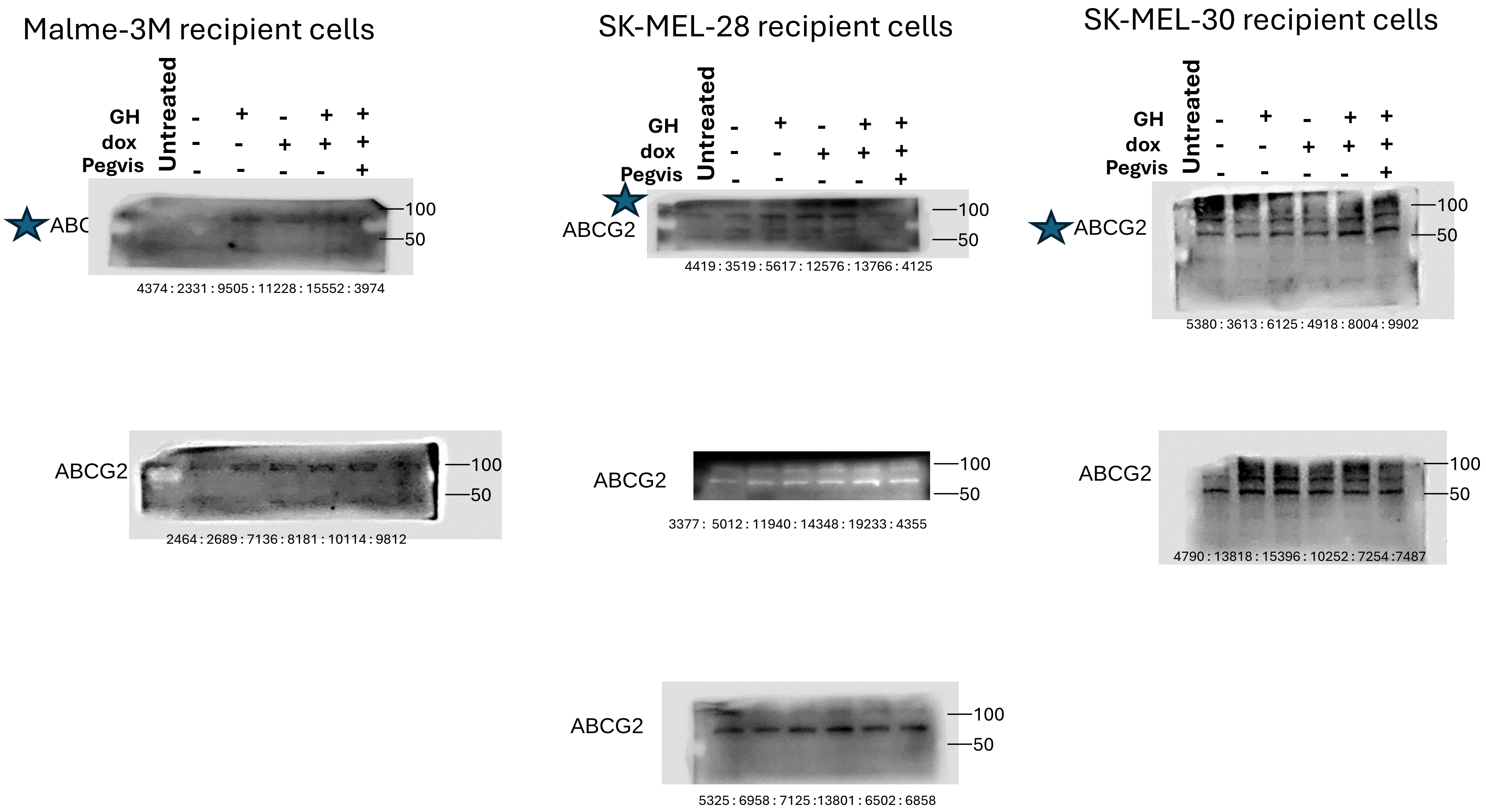

Figure 2. GH elevates the expression of ABC-transporter pumps in tumor-derived exosomes and their transfer to recipient cells

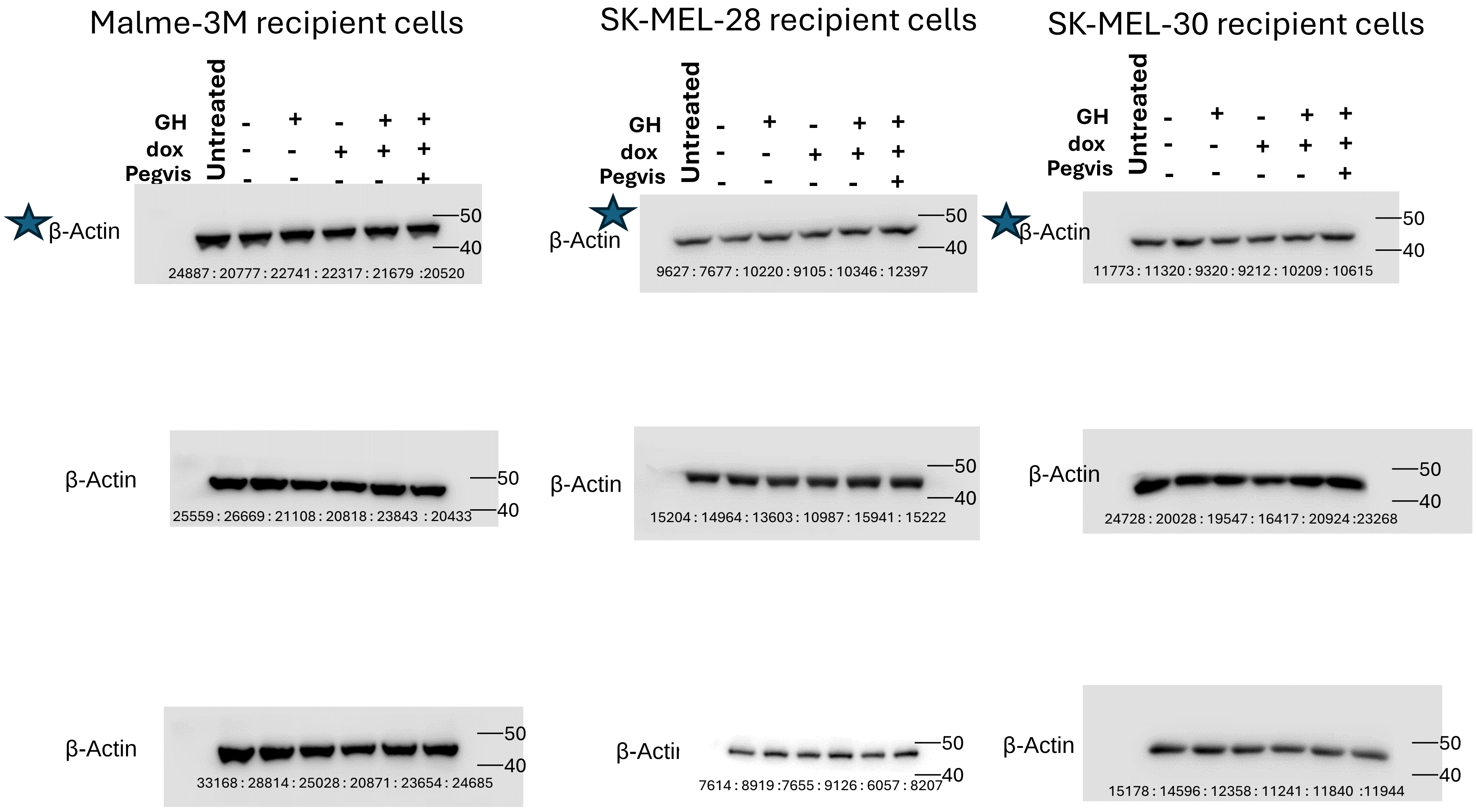

Figure 3: Local action of GH modulates expression of exosomal ABC transporters

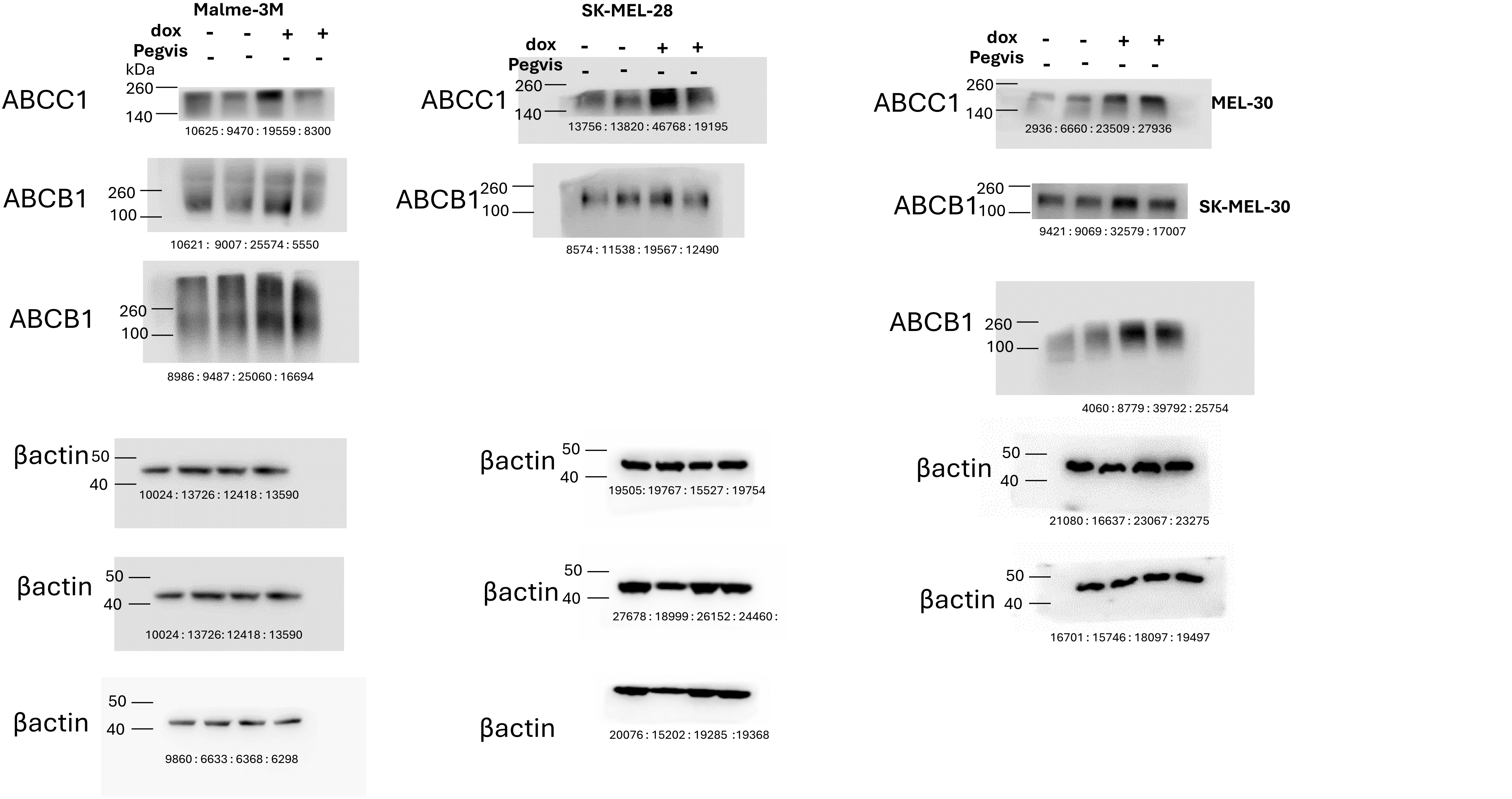

Figure 4. Pegvisomant attenuates melanoma cell migration via exosomes

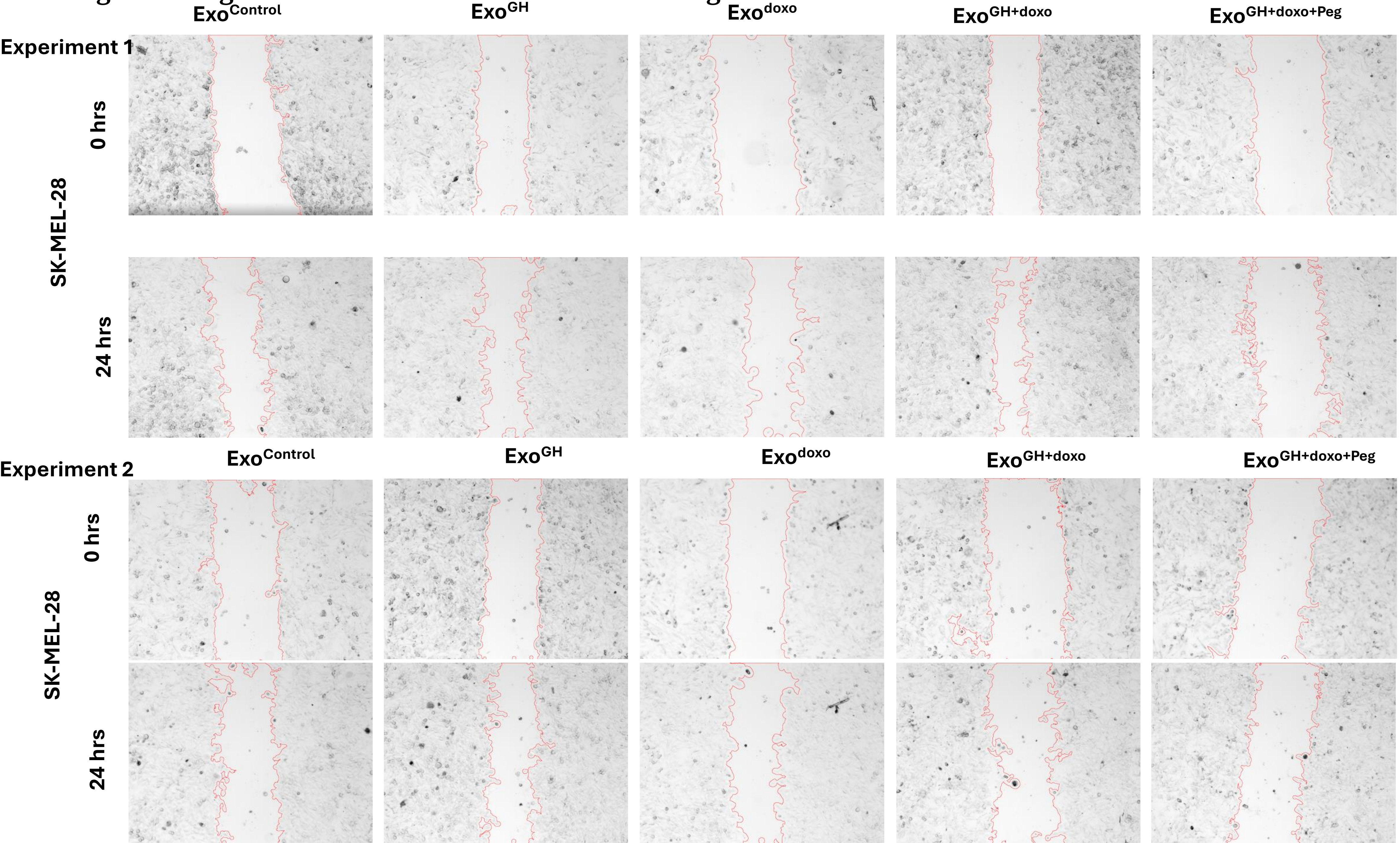

Figure 4. Pegvisomant attenuates melanoma cell migration via exosomes

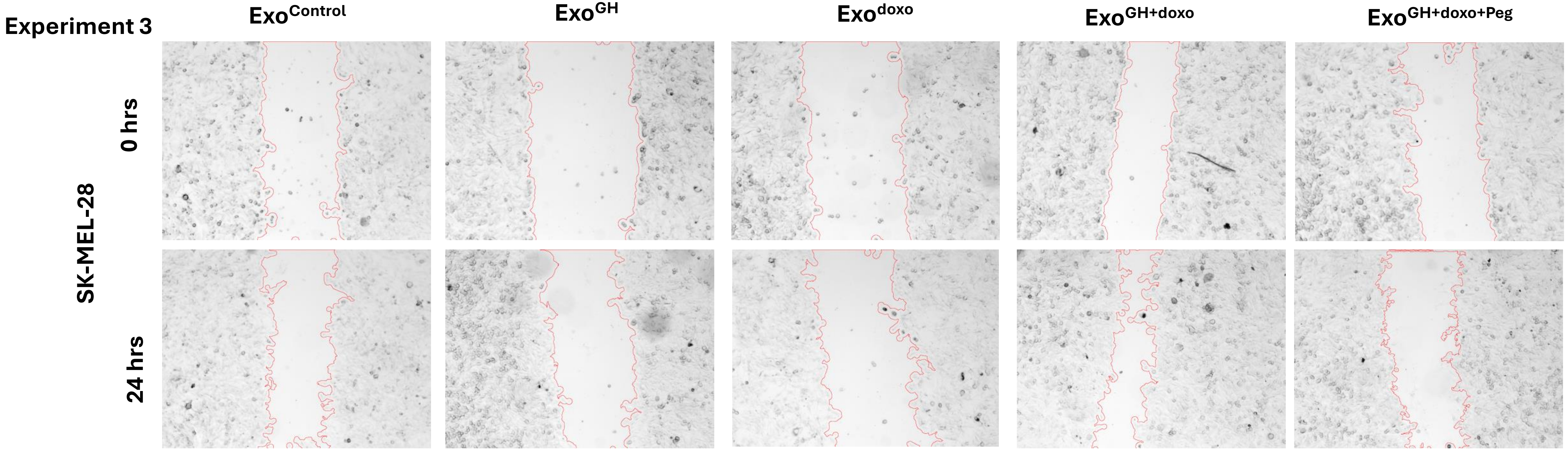

Figure 4. Pegvisomant attenuates melanoma cell migration via exosomes

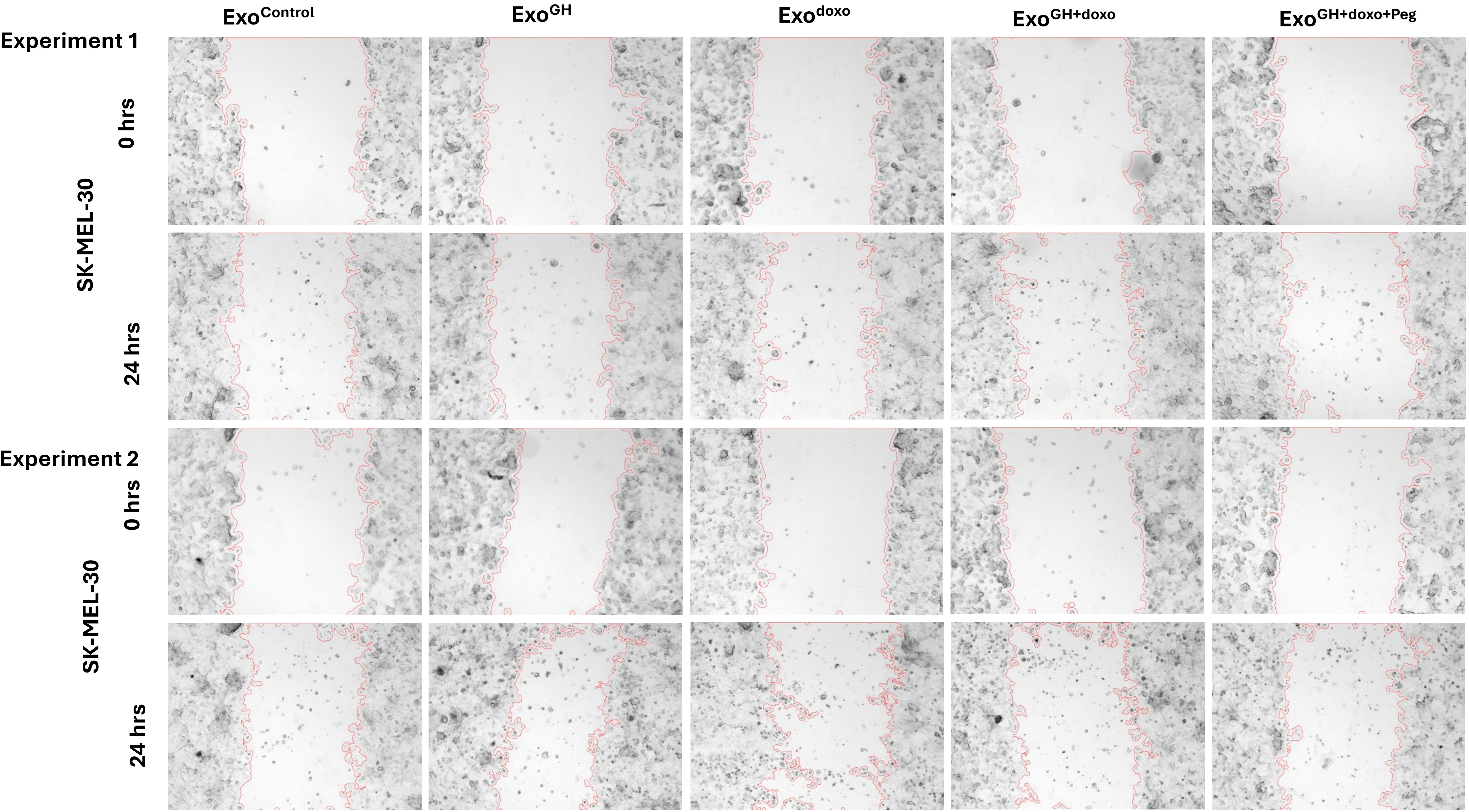

Figure 4. Pegvisomant attenuates melanoma cell migration via exosomes

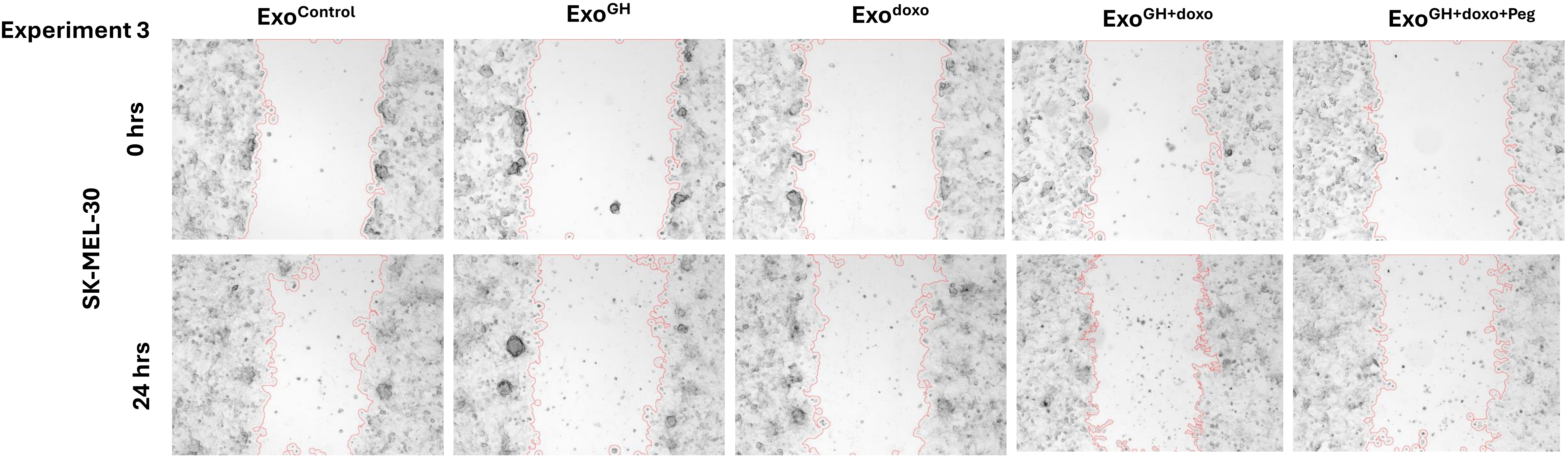

Figure 5. GH elevates N-cadherin and MMP2 in melanoma-derived exosomes and melanoma cells receiving the exosomes

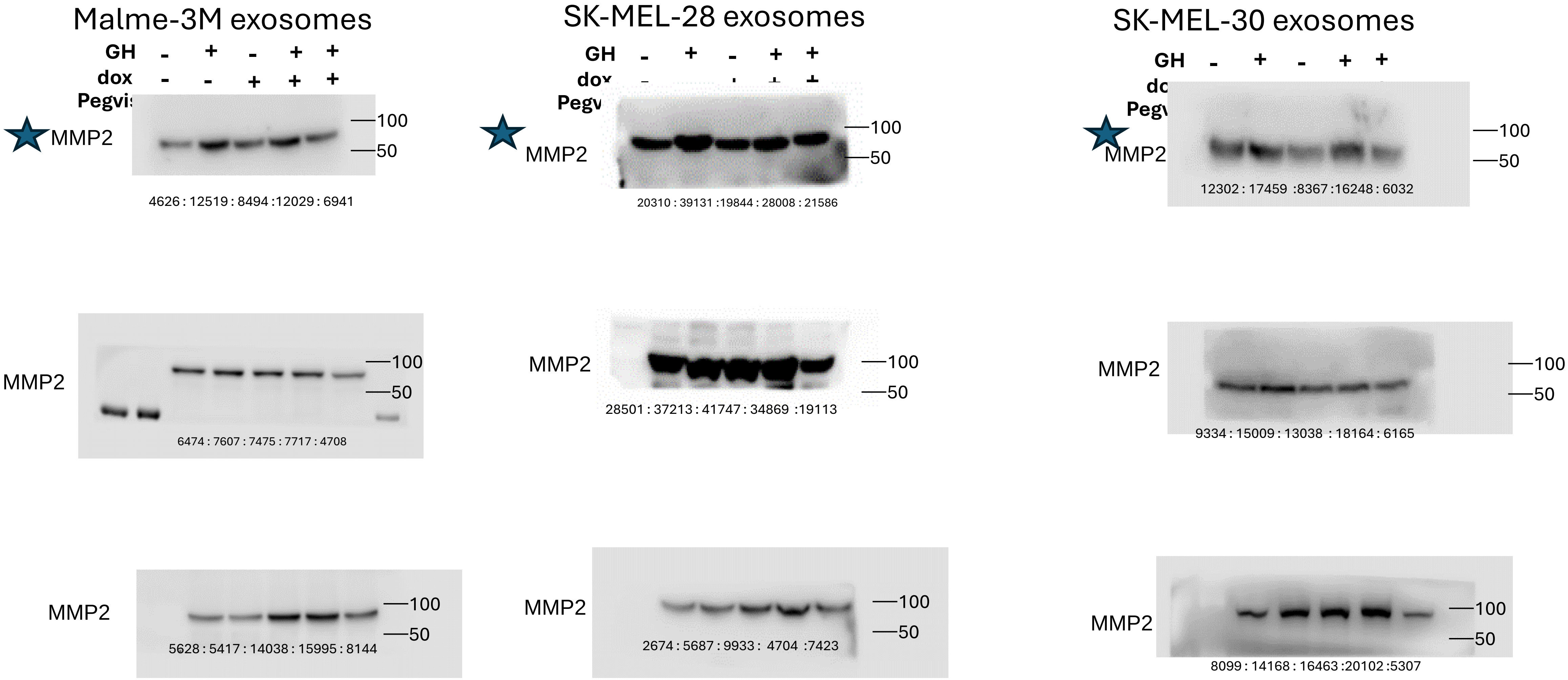

Figure 5. GH elevates N-cadherin and MMP2 in melanoma-derived exosomes and melanoma cells receiving the exosomes

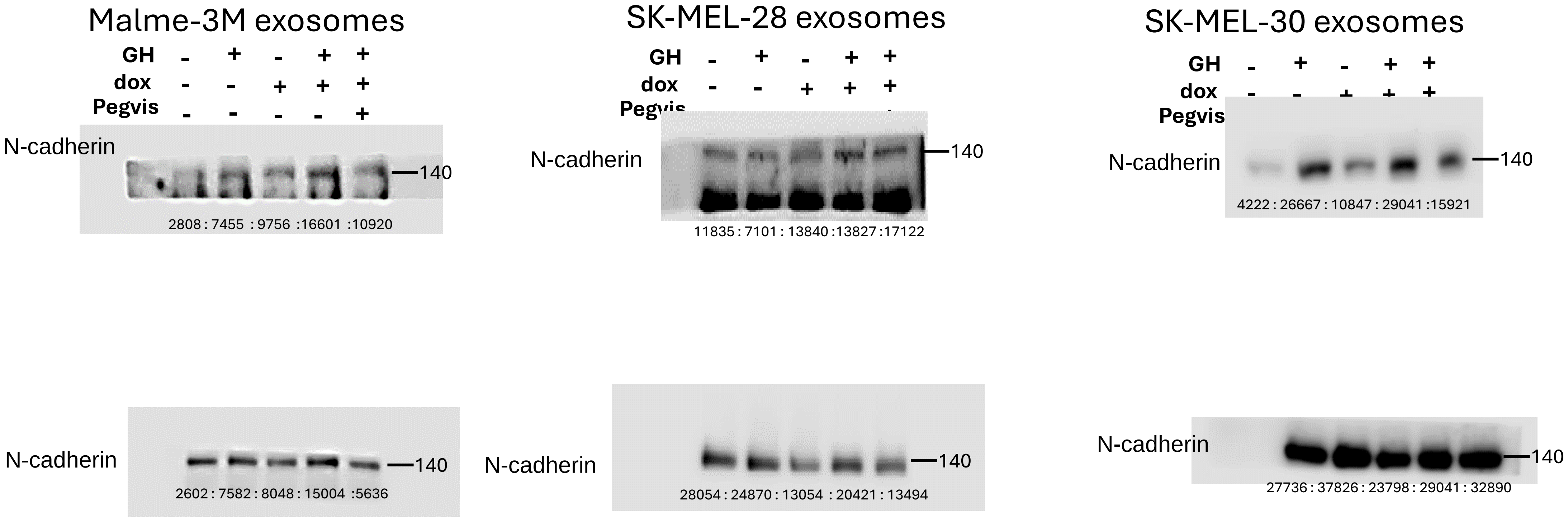

Figure 5. GH elevates N-cadherin and MMP2 in melanoma-derived exosomes and melanoma cells receiving the exosomes

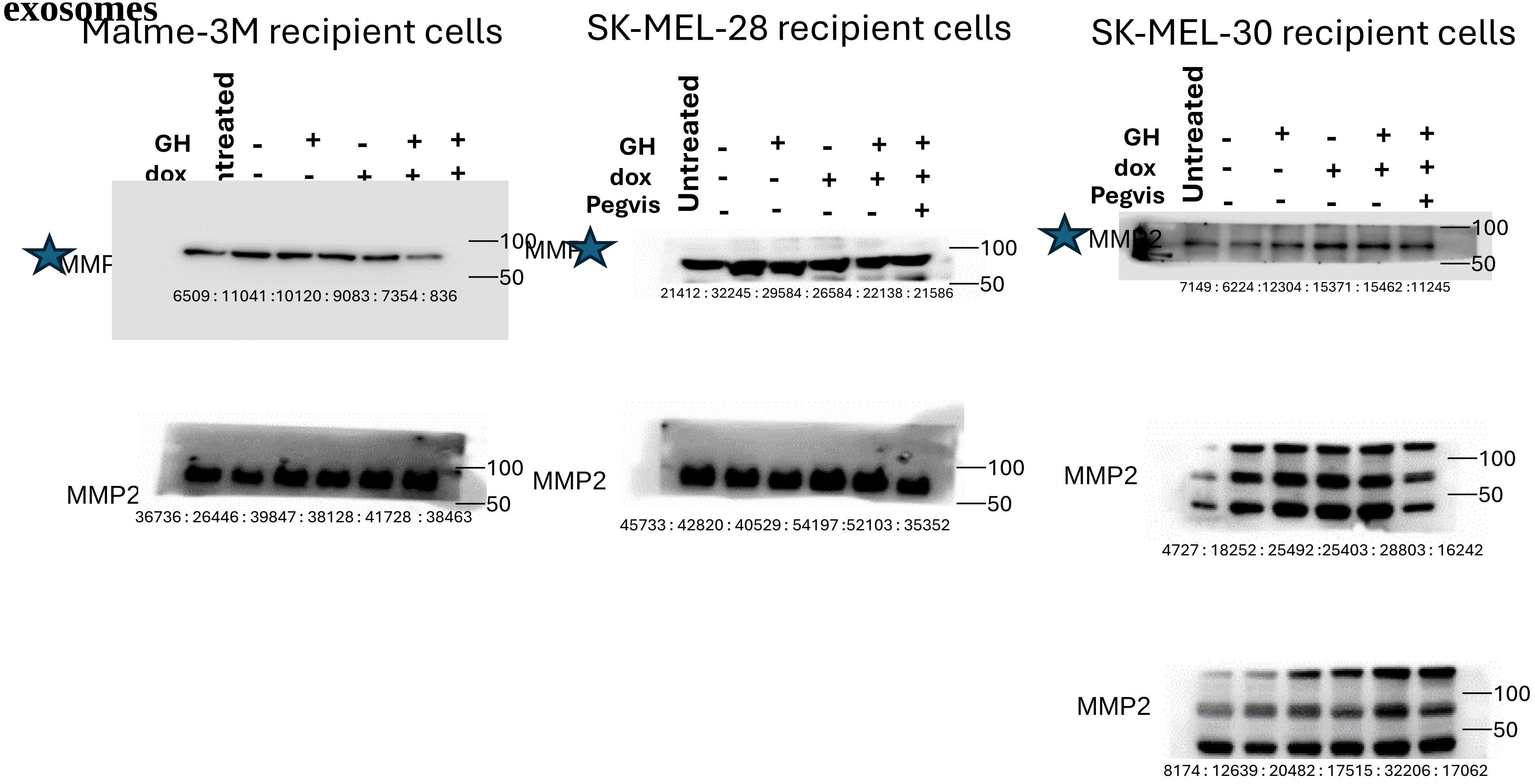

Figure 5. GH elevates N-cadherin and MMP2 in melanoma-derived exosomes and melanoma cells receiving the exosomes

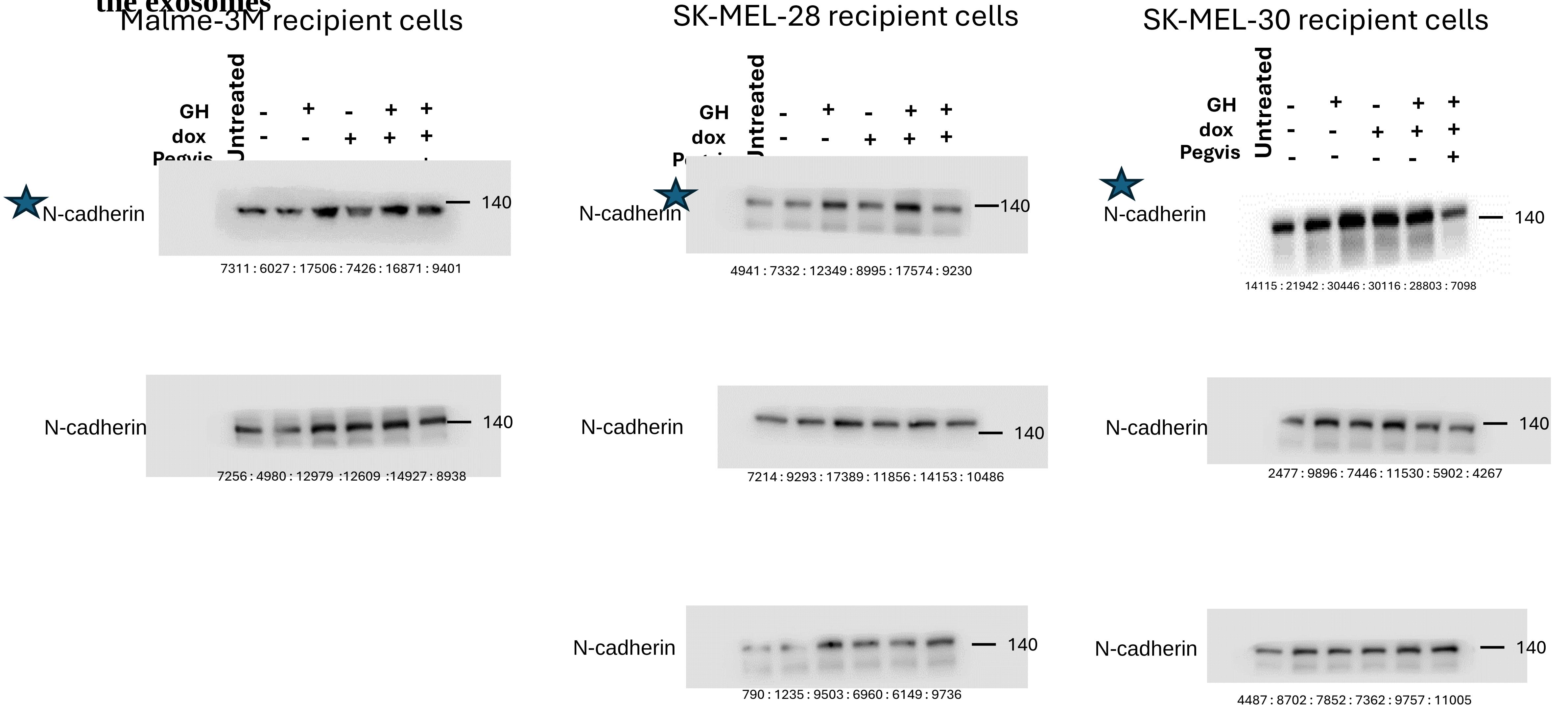

Supplementary Figure 3. GH elevates the expression of ABC-transporter pumps melanoma cells

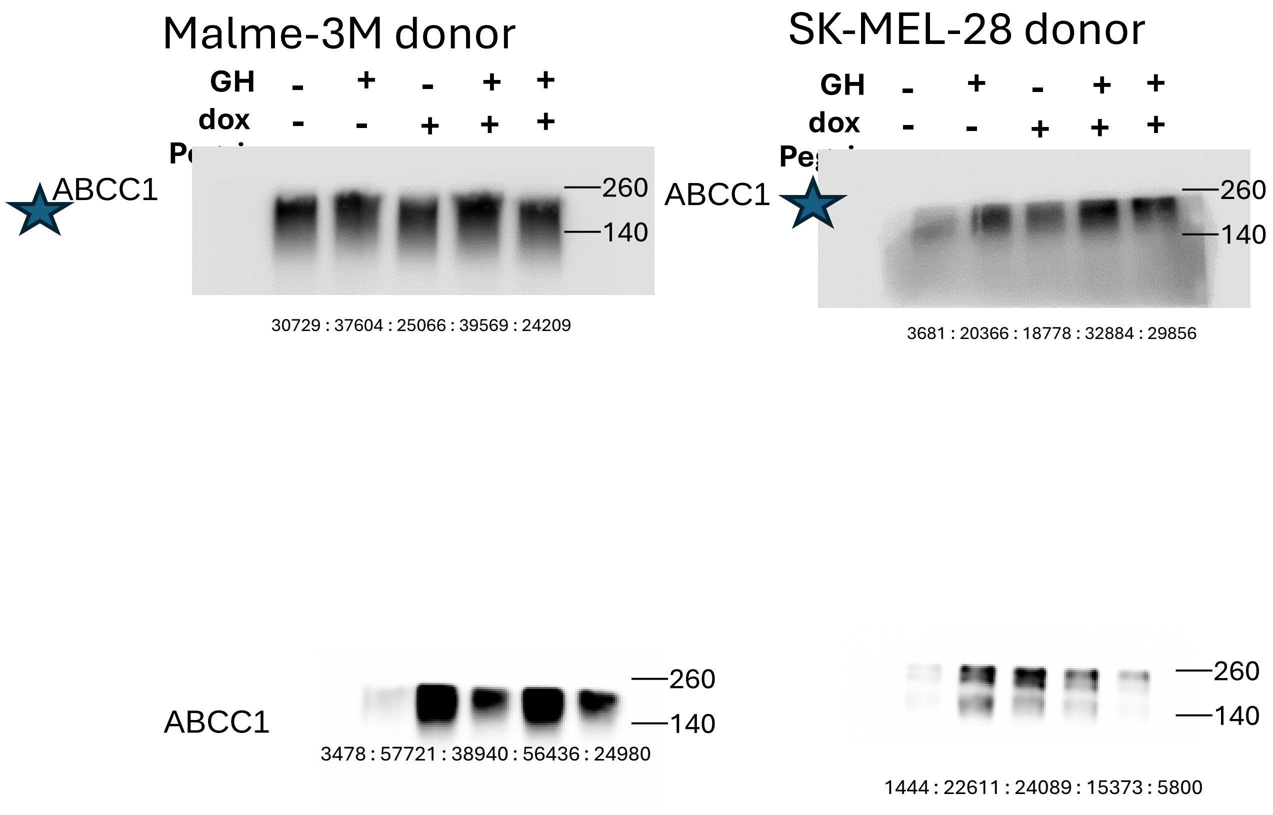

Supplementary Figure 3. GH elevates the expression of ABC-transporter pumps melanoma cells

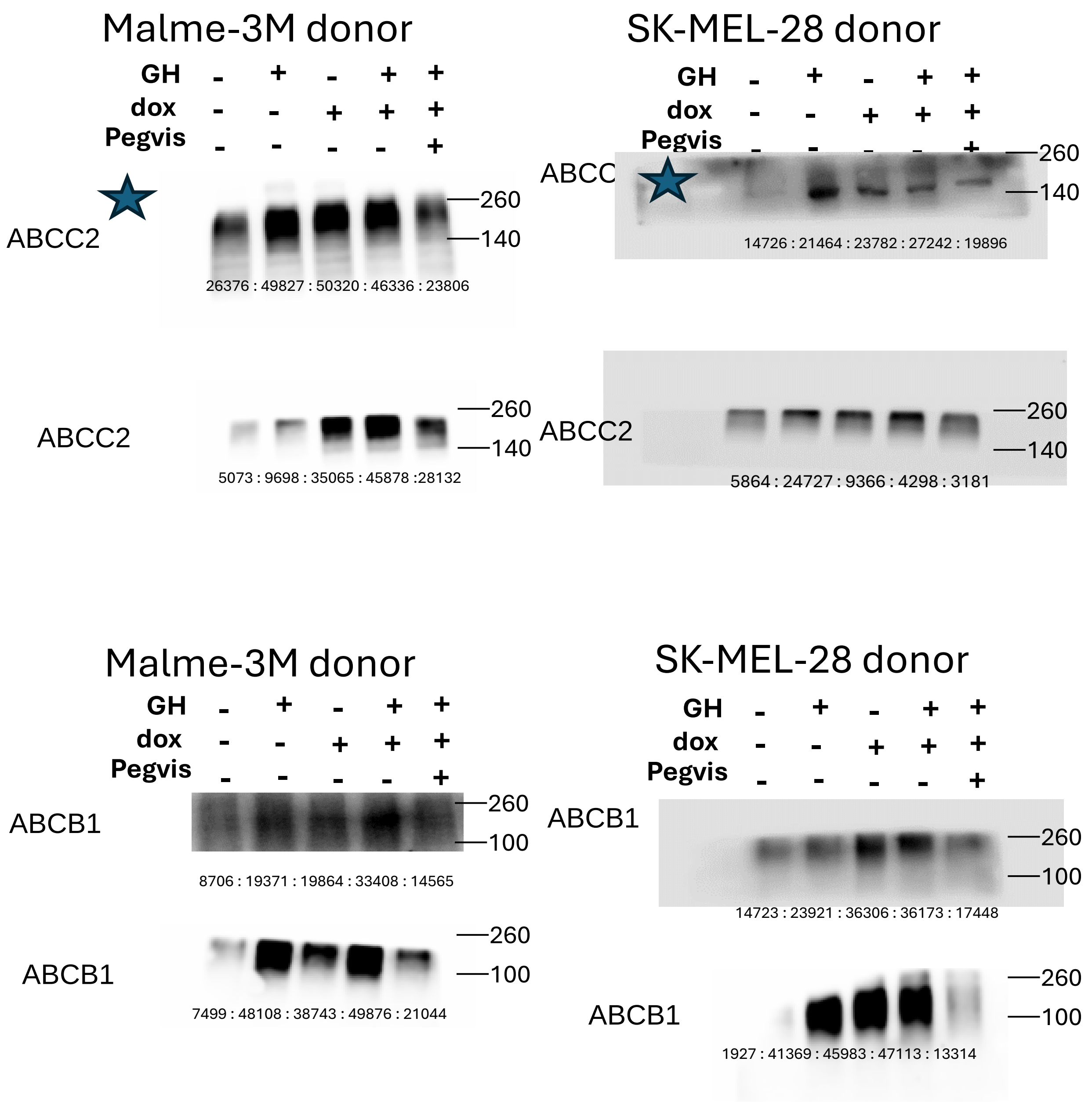

Supplementary Figure 3. GH elevates the expression of ABC-transporter pumps melanoma cells

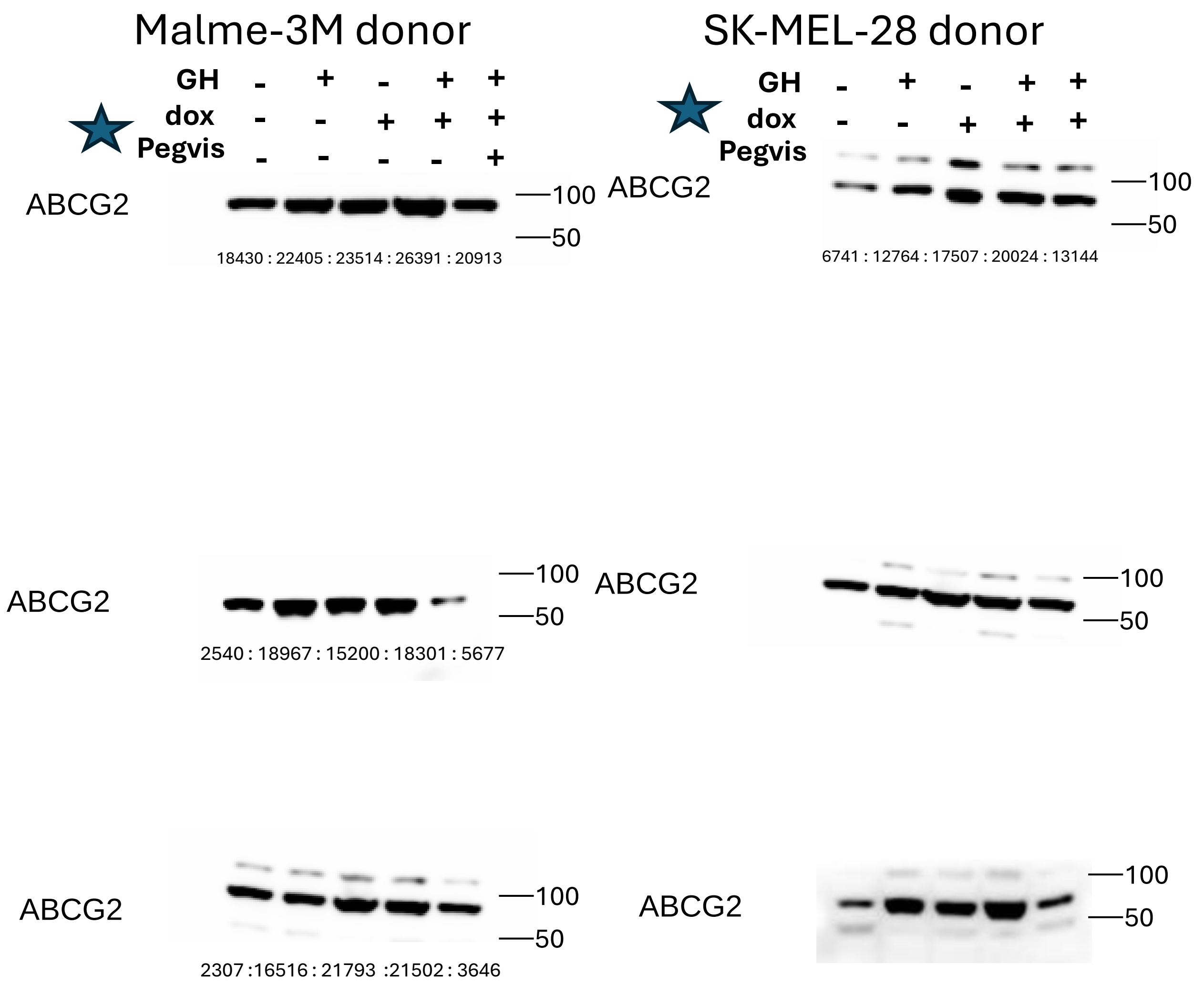

Supplementary Figure 5. GH elevates the expression of N-cadherin and MMP2 in melanoma cells

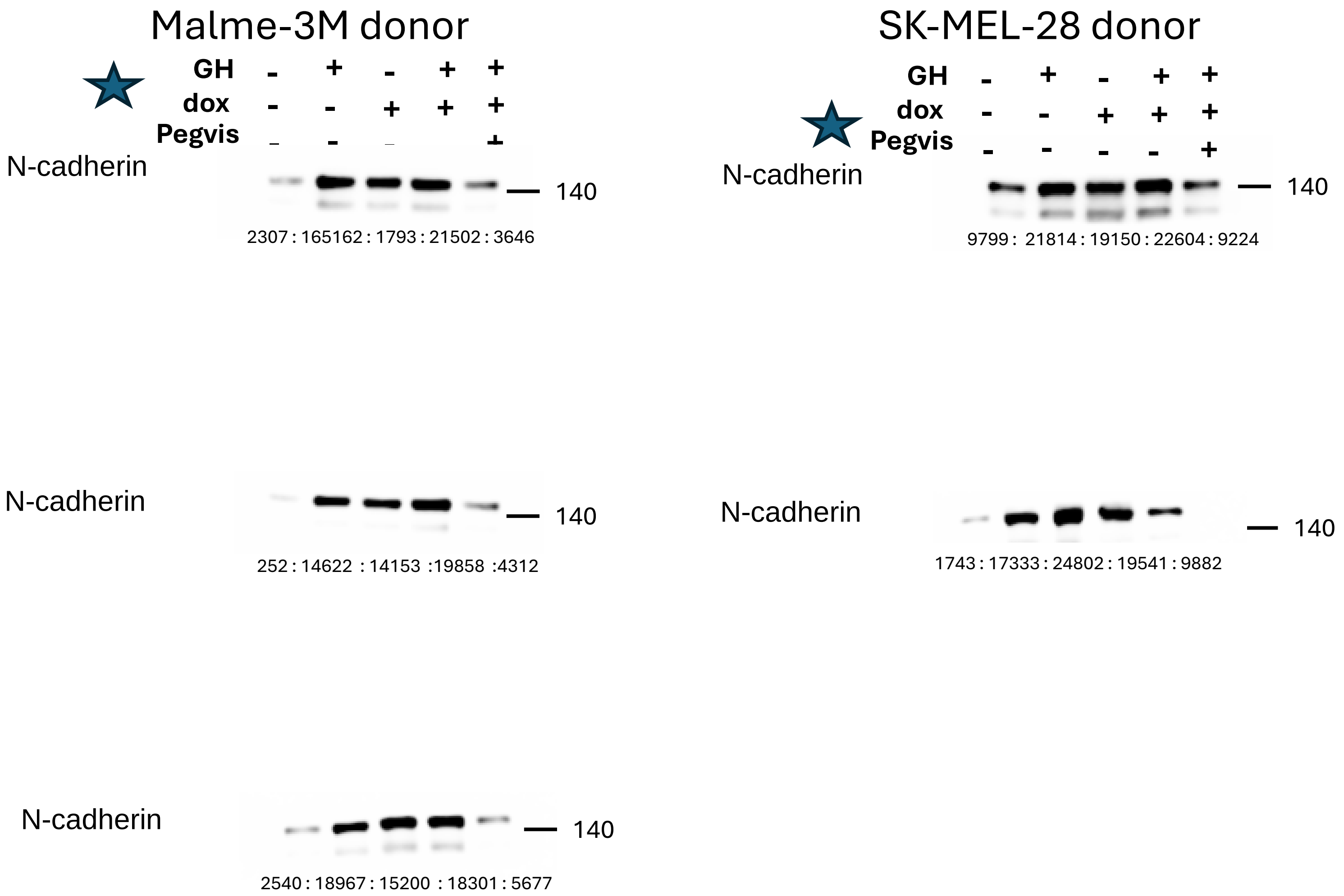

Supplementary Figure 5. GH elevates the expression of N-cadherin and MMP2 in melanoma cells

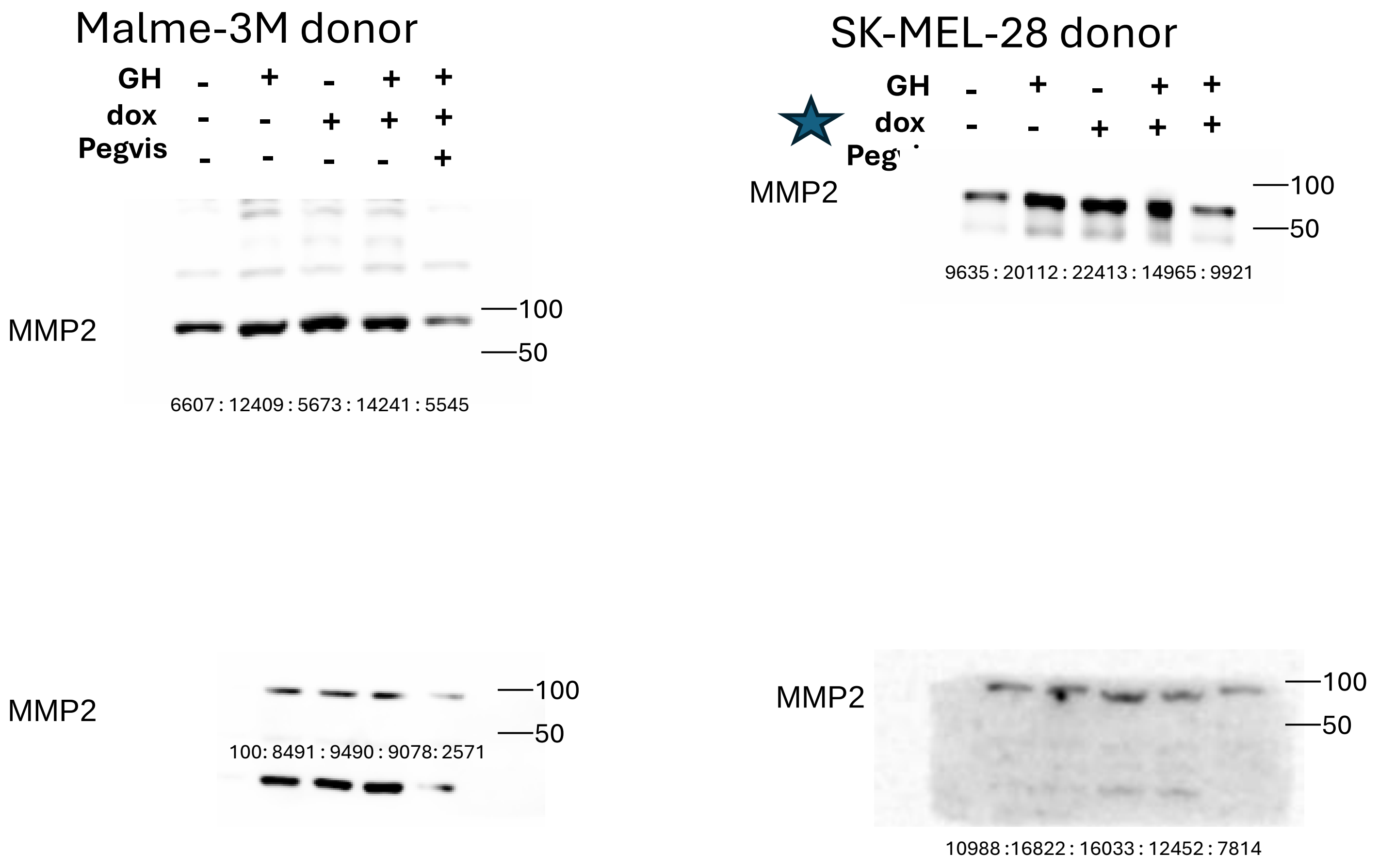

Supplementary Figure 6.

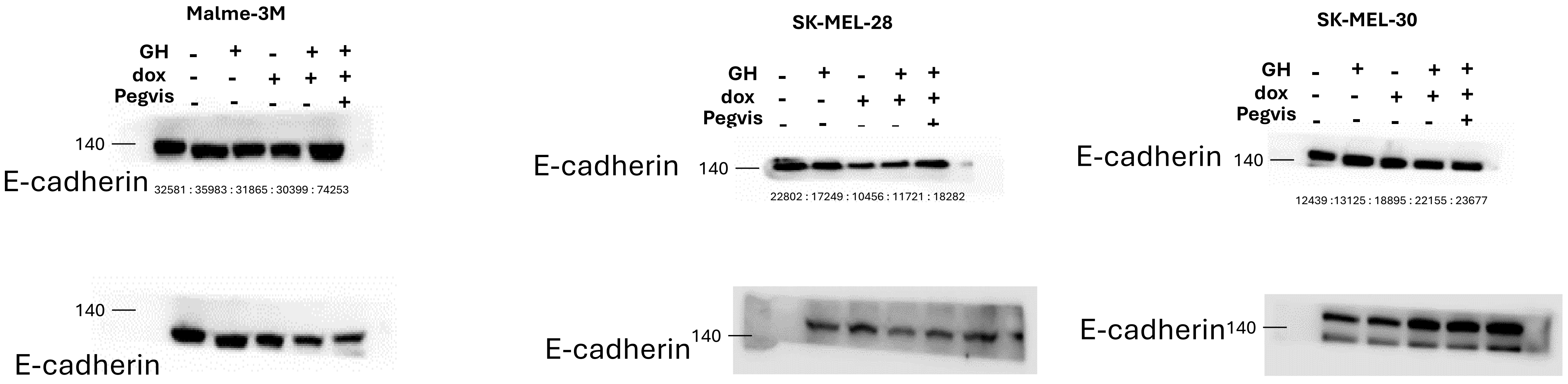

Supplementary Figure 6.

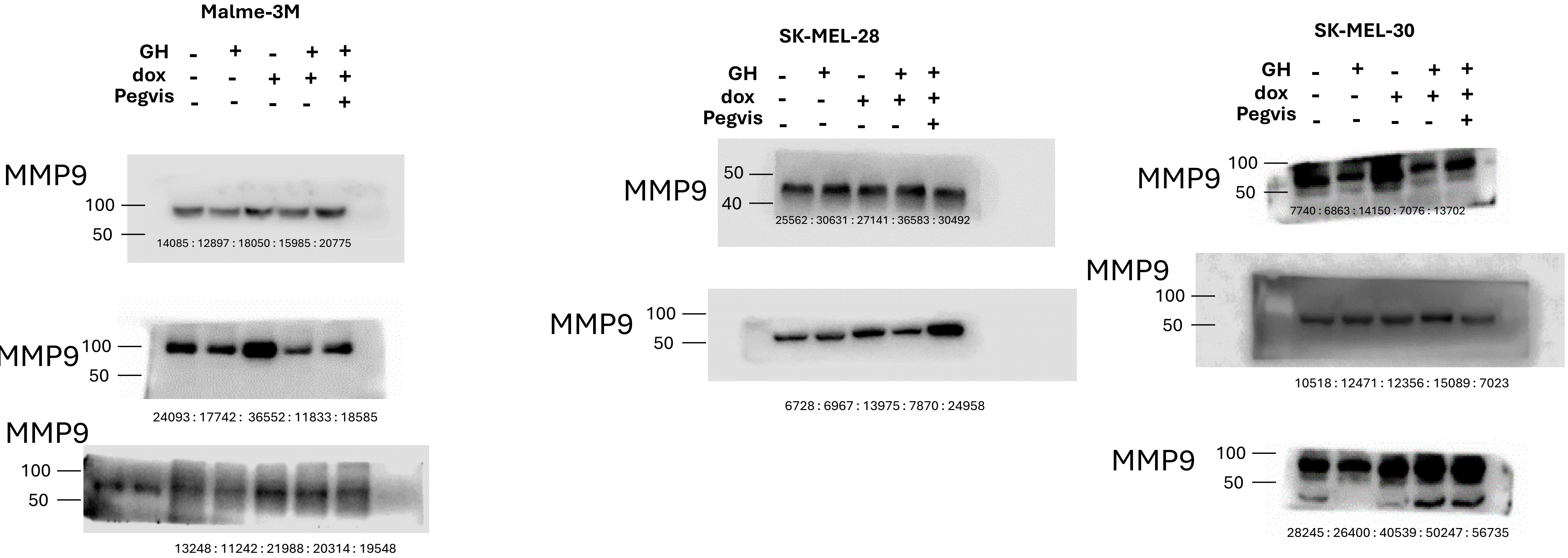

Supplement: Supplementary file 1 [file cancers-16-02636-s001.zip › cancers-3099853-supplementary/Raw and Repeats data.pdf]
